# Through-Layer Contact with Encapsulated Graphene Using One-Step Laser Scribing

**DOI:** 10.1021/acsami.5c09559

**Published:** 2025-08-05

**Authors:** Sang-Chan Park, Won Gyun Park, Chulsoo Kim, Jae-Hyuk Ahn

**Affiliations:** Department of Electronics Engineering, 26715 Chungnam National University, Daejeon 34134, Republic of Korea

**Keywords:** encapsulated graphene, laser-induced graphene, through-layer contact, multilayered contact, field-effect transistor, sodium sensor

## Abstract

Encapsulated graphene plays a key role in enabling stable and functional operation of next-generation electronic and sensing devices. However, establishing electrical contact with encapsulated graphene remains a significant challenge because of the need for separate etching and metal deposition steps, which require complex multistep fabrication processes. This paper introduces a scalable one-step method for forming electrical contacts with encapsulated graphene using CO_2_ laser scribing. This approach simultaneously etches the encapsulation layer and forms laser-induced graphene (LIG), enabling direct contact with the underlying graphene in a single step under ambient conditions. The formation of a through-layer LIG contact was experimentally verified via real-time electrical monitoring during laser scribing. This technique reliably creates electrical contacts using various organic and inorganic encapsulation layers, demonstrating broad material compatibility. Using this method, we demonstrated multilayered electrical contacts, top- and bottom-gate graphene field-effect transistors (FETs) with through-layer LIG source/drain electrodes, and sodium-selective sensors in both electrochemical and FET configurations. The proposed method simplifies fabrication and enables the low-cost, scalable integration of encapsulated graphene into next-generation electronic and sensing applications.

## Graphical abstract

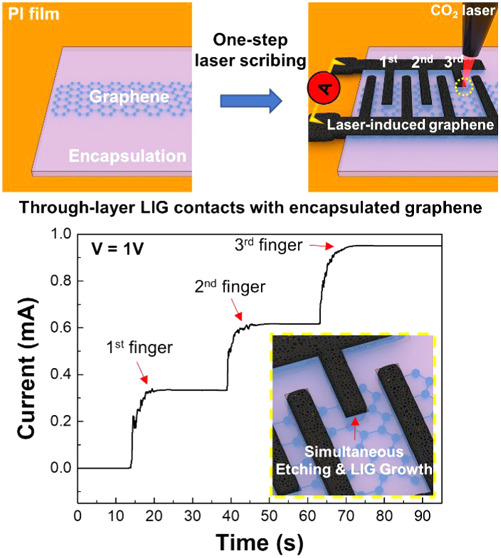

## Introduction

Graphene, a monolayer of carbon atoms in a honeycomb lattice with sp^2^ bonding, is a promising material for active and channel applications owing to its high electrical conductivity,^1–3^ mechanical strength,^4,5^ and optical transmittance.^6–8^ High-quality, large-area graphene synthesized by chemical vapor deposition (CVD)^9,10^ can be integrated into field-effect transistors (FETs),^11–13^ biosensors,^14–16^ chemical sensors,^17,18^ and flexible electronics.^19–21^ In addition, CVD-grown graphene can be transferred onto diverse substrates via wet or dry methods,^22–25^ with roll-to-roll processes offering scalable, large-area transfer characteristics.^26,27^ These properties have supported the development of lightweight, flexible, and multifunctional electronics. Encapsulation is commonly employed to enhance the environmental stability and device integration of graphene. Encapsulated graphene with gate dielectrics exhibits bipolar transfer characteristics because of its Dirac cone-shaped band structure,^28,29^ and has also been used in chemical and biological sensors,^14,18^ wherein functional sensing layers are placed on top of graphene to modulate its electrical properties under analyte exposure. This encapsulation protects graphene from environmental degradation and supports the construction of stable devices.^30–32^

Electrical contact with encapsulated graphene typically involves multiple processing steps, including encapsulation etching and metal deposition via conventional photolithography, reactive-ion etching (RIE), and metal evaporation.^33,34^ In most cases, first, contact is established between graphene and metal via evaporation, followed by applying an encapsulation process and opening the contact pads.^35,36^ Pristine graphene, lacking surface bonding sites, shows a high contact resistance with metals. To address this issue, the contact region is often patterned to increase the exposure of graphene edge, thereby enhancing electrical performance despite a reduction in overall contact area.^37^ Various strategies have been explored to establish reliable electrical contact, including via contacts^38^ and edge contacts.^34,39^ In hexagonal boron nitride (h-BN)-encapsulated graphene, electron-beam lithography, O_2_ and CF_4_ plasma etching, and metal evaporation have been utilized.^33^ Partially fluorinated graphene serves as both a protective layer and an etch-stop layer during SF_6_ plasma etching of h-BN,^40^ enabling effective metal deposition for contact formation. Additionally, edge contact has been demonstrated in Parylene-C-encapsulated graphene via O_2_ plasma etching and subsequent metal deposition.^41^ However, despite these advancements, conventional methods remain limited by their complexity, dependence on multiple tools, and low compatibility with scalable fabrication techniques. This limitation is attributed to the requirement of encapsulation-layer etching followed by metal deposition for forming electrical contacts—two fundamentally opposing processes, whose efficient integration is challenging.

CO_2_ lasers are widely used in industries for drilling, cutting, and surface modification because of their low costs, high efficiency, and ambient operating conditions.^42,43^ Laser scribing of polyimide (PI) produces conductive laser-induced graphene (LIG),^44^ which has been explored for applications in energy storage, flexible electronics, and chemical sensing.^44–48^ Its compatibility with direct patterning, scalable processing, and flexible substrates facilitates integration of conductive contacts into encapsulated graphene devices.

In this study, we demonstrate a simple one-step method for forming electrical contacts with encapsulated graphene using laser scribing. CO_2_ laser irradiation of encapsulated graphene on a PI substrate etches the encapsulation layer to expose the graphene and simultaneously forms a 3D porous carbon-based conductor on the PI substrate, known as LIG^44,46,48–51^ which makes ohmic contact with the encapsulated graphene. Our approach, which is based on a through-layer LIG contact, simplifies the contact process by integrating etching and metal formation into a single-step laser-scribing process. We experimentally optimized the CO_2_ laser conditions to efficiently etch the encapsulation layer and grow LIG in direct contact with a graphene layer. This through-layer LIG contact method is applicable to various encapsulation materials, including organic and inorganic layers. Building on this approach, multilayered electrical contacts were realized by repeatedly transferring the CVD-grown graphene with encapsulation layers, followed by CO_2_ laser irradiation to create LIG contacts that effectively connected each graphene layer. This technique was used to implement graphene transistors with through-layer LIG source/drain electrodes, fabricated using a one-step CO_2_ laser-scribing process, achieving a performance comparable to that of conventional multistep fabrication methods. Furthermore, the through-layer approach has been extended to electrochemical and electrical sodium sensors, where the encapsulation layer functions as a chemical sensing layer, whereas LIG electrodes enable efficient electrical signal extraction.

## Results and Discussion

### LIG for Electrical Contact Through Encapsulation Layers

The conventional process for establishing electrical contact with encapsulated graphene involves multiple steps and is thus both time-consuming and complex (Figure 1a­(i)). Typically, this process requires photolithography followed by etching of the top encapsulation layer to expose the underlying graphene. After patterning and etching the encapsulation layer, metal deposition or printing was performed using an evaporator or printing machine to form an electrical contact with the graphene layer. However, this approach requires precise alignment and sequential processing steps, increasing the fabrication time and requiring specialized equipment.

**Figure 1:**
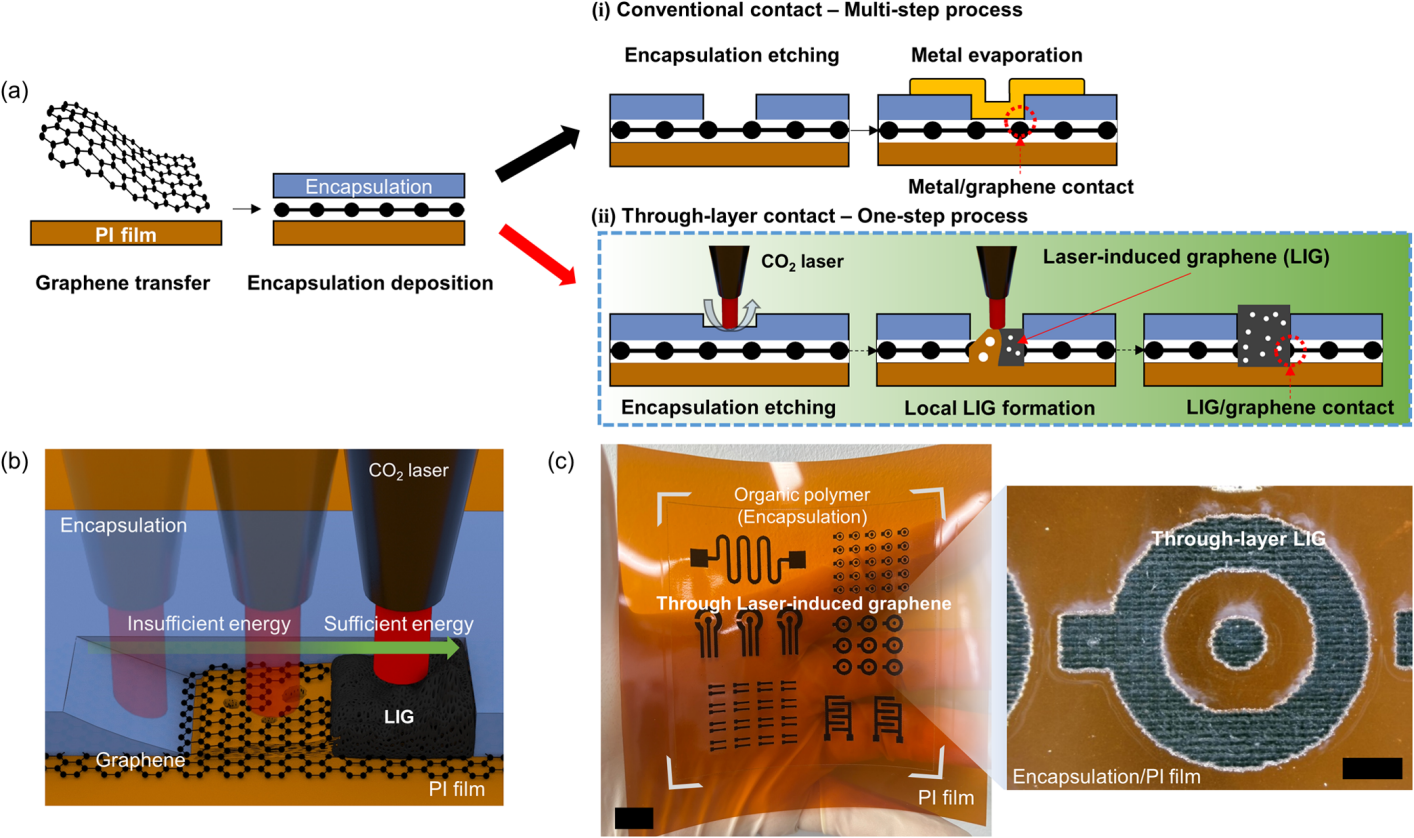
Electrical contacts to encapsulated graphene using LIG formed via CO_2_laser scribing. (a) Contact methods for encapsulated graphene: (i) conventional multistep process involving separate etching and metal deposition and (ii) proposed one-step through-layer contact method that simultaneously etches the encapsulation layer and forms an LIG electrode. (b) 3D schematic illustrating CO_2_ laser irradiation of the encapsulated graphene/PI film under different laser energy conditions. (c) Optical images of the fabricated LIG electrodes: a large-area PI film with various electrode patterns formed through an organic polymer encapsulation layer (left) and a magnified view of a pair of circular electrodes (right). Scale bars: 10 (left) and 1 (right) mm.

To address the limitations of conventional contact methods, we propose a through-layer LIG contact approach using CO_2_ laser scribing, which enables direct contact formation in a single-step process (Figure 1a­(ii)). Because the two carbon-based materials employed in this study serve fundamentally different roles, clarifying their functions is crucial. CVD-grown graphene, with its atomically thin structure and field-effect modulation capability, serves as the active channel material, whereas LIG, generated via CO_2_ laser irradiation, acts as a porous, conductive electrode formed directly through the encapsulation layer. The simplicity of the CO_2_ laser-scribing process, which does not require vacuum conditions or complex photolithography steps, makes it highly attractive for scalable and cost-effective fabrication of electronic devices. When a CO_2_ laser is irradiated onto an encapsulated graphene/PI film, three key phenomena occur simultaneously: (1) the encapsulation layer is etched away; (2) the PI film swells and forms pores; and (3) full LIG formation occurs, establishing direct contact with the graphene layer. This one-step laser-based method offers a significantly faster and more efficient alternative to conventional metal–graphene contact fabrication.

The importance of laser energy control in this process is illustrated in Figure 1b, which presents a 3D schematic of the CO_2_ laser irradiation at different energy levels. When the laser energy was insufficient, only the encapsulation layer was partially etched, preventing full contact with the underlying graphene. However, with sufficient laser energy, the through-layer LIG fully forms and establishes a direct electrical contact with the encapsulated graphene in a single step. This simultaneous etching and contact formation capability highlight the efficiency of CO_2_ laser scribing compared with conventional methods.

Figure 1c shows optical images of the fabricated LIG electrodes formed through the encapsulation layer on a large-area PI film (10 cm × 10 cm), demonstrating the scalability of this approach. The electrodes exhibit various geometries, highlighting the versatility of this fabrication method. The magnified image of a pair of circular LIG electrodes further confirmed the structural integrity and precision of the fabricated electrodes.

### Real-Time Verification of Through-Layer LIG Contact Formation

The *in situ* through-layer LIG contact with the encapsulated graphene was confirmed by monitoring the electrical current in real time. An interdigitated electrode structure was selected for the measurements. Briefly, bilayer graphene was transferred onto a PI film using the thermal-release tape (TRT) method and encapsulated in polyvinyl chloride (PVC) via a bar coater. One pair of interdigitated through-layer LIG electrodes was first formed by CO_2_ laser scribing with the electrode fingers straddling the graphene. Subsequently, a second pair of interdigitated through-layer LIG electrodes were fabricated for real-time current monitoring. Figure 2a illustrates the schematic procedure. Figure 2b and Movie S1show that no current initially flowed because of the absence of contact with the encapsulated graphene layer. However, over time, the current began to flow as the through-layer LIG made contact with graphene owing to sufficient laser energy. The variation in current step sizes is attributed to nonuniform contact between LIG and graphene, which may arise from incomplete graphene transfer, residues from the TRT process, or surface roughness in the encapsulation layer introduced during bar coating.

**Figure 2:**
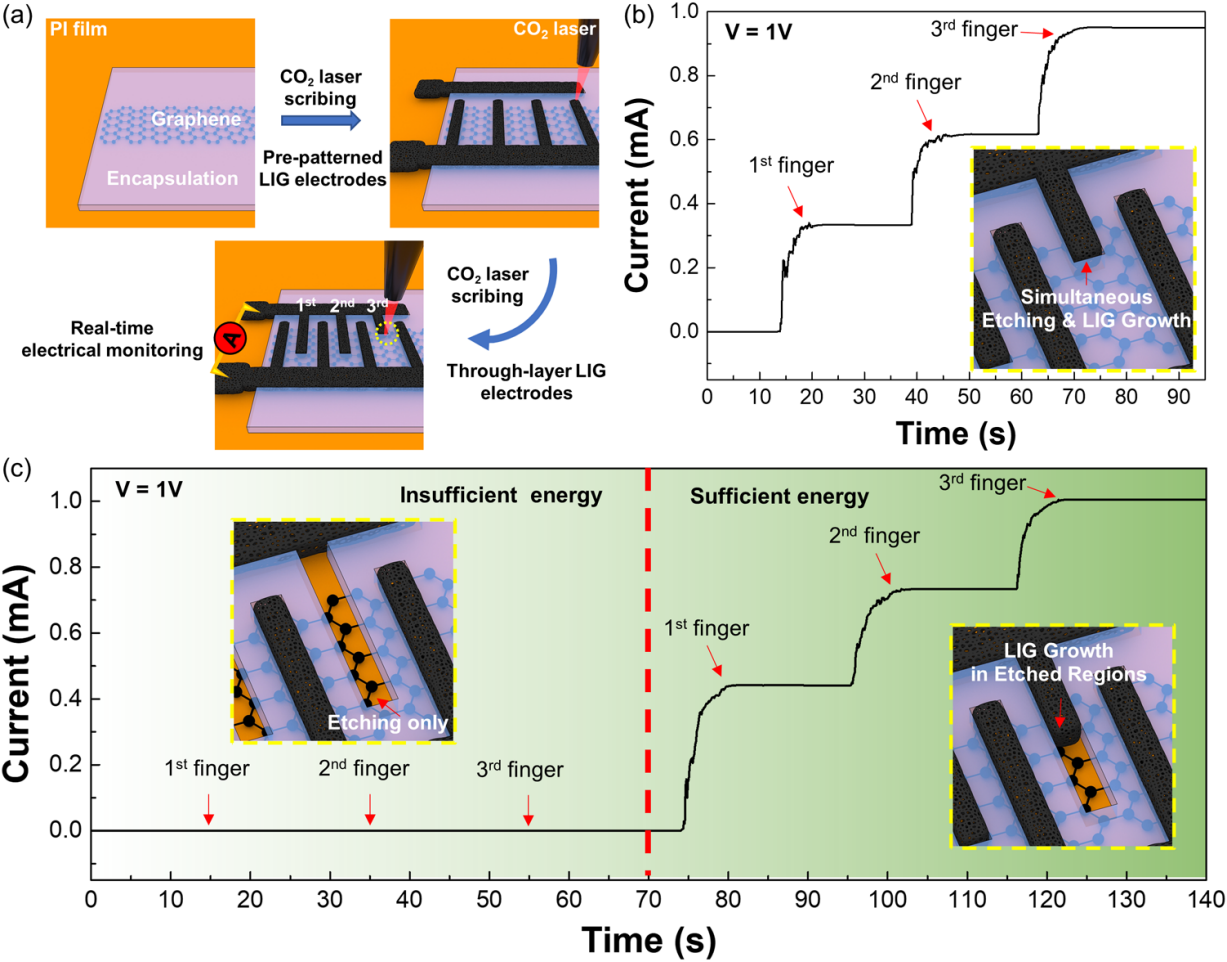
Real-time electrical monitoring of through-layer LIG contact formation in encapsulated graphene. (a) Schematic of real-time electrical measurements using *in situ* through-layer LIG electrodes fabricated by CO_2_ laser scribing. (b) Real-time electrical measurements of through-layer LIG electrodes fabricated by CO_2_ laser scribing at sufficient laser energy, which simultaneously etched the encapsulation layer and formed an LIG electrode. (c) Real-time electrical current measurement of the *in situ* interdigitated through-layer LIG electrodes fabricated using the same procedure as in (b), except for the CO_2_ laser-scribing conditions. Initially, a CO_2_ laser with insufficient energy was applied to the three-electrode fingers, etching only the encapsulation layer without forming electrical contact with the graphene channel. Subsequently, the same fingers were irradiated with sufficient laser energy to form the through-layer LIG electrodes. The insets show schematics of the finger regions under different CO_2_ laser-scribing conditions.

To further investigate contact formation, interdigitated through-layer LIG electrodes were fabricated under varying laser conditions. Initially, the PVC-encapsulated graphene was irradiated with insufficient CO_2_ laser energy (3.79 J/cm^2^), which etched only the encapsulation layer without forming through-layer LIG fingers (Figure 2c and Movie S2). The same electrode fingers were then reirradiated with sufficient laser energy (8.16 J/cm^2^), resulting in current flow owing to the successful formation of a through-layer LIG contact with the graphene channel (Figure 2c). The fluence calculation is described in the Supporting Information (eq S1). Notably, even when the encapsulation layer was etched with insufficient energy, the underlying graphene remained undamaged, and a through-layer LIG contact was successfully formed through subsequent CO_2_ laser scribing.

### Optimization of Laser Parameters and Analysis of Encapsulation Layer Thickness Effects

As mentioned previously, sufficient laser energy is required to simultaneously etch the encapsulation layer and form the LIG electrode. To validate the through-layer LIG contact with the encapsulated graphene and optimize the laser-scribing parameters, a CO_2_ laser was irradiated onto an encapsulation layer covering the graphene, and the electrical conductivity was measured between the through-layer LIG electrodes in contact with the graphene layer (Figure 3a). PVC, a commonly used passivation material and matrix for ion-selective membranes, was selected as the representative encapsulation layer. After coating PVC onto the graphene, CO_2_ laser ablation was performed using various laser parameters to determine the optimal conditions for high-conductance LIG formation (Figure S1). At a fixed PVC thickness, PVC-encapsulated graphene was irradiated using a CO_2_ laser while varying the scan rate. At a high scan rate, incomplete through-layer LIG formation was observed, likely owing to energy dissipation during PVC etching, resulting in low or negligible conductivity. As the scan rate decreased, fully formed through-layer LIG structures exhibiting high conductivity were obtained.

**Figure 3:**
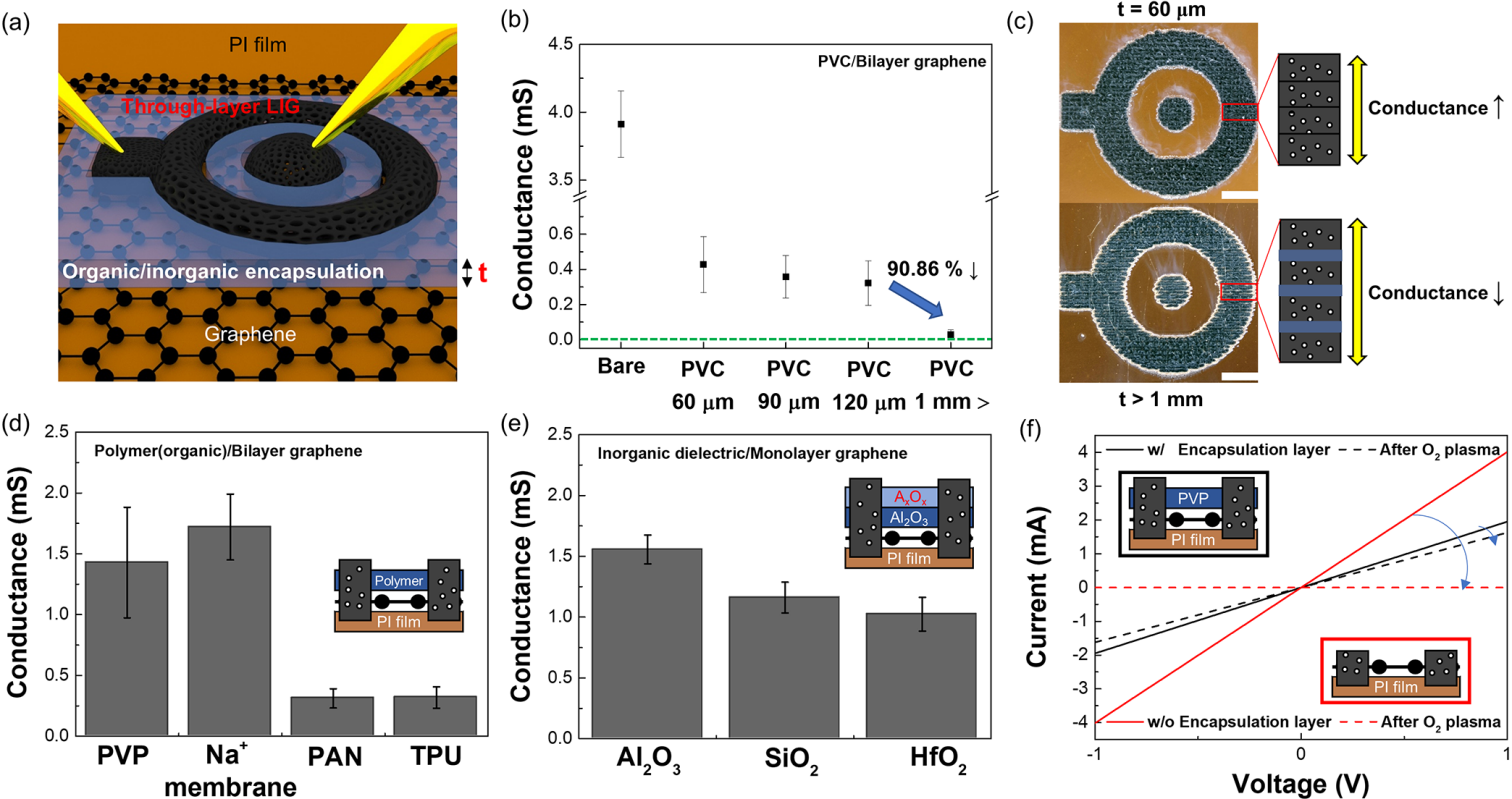
Electrical characterization of through-layer LIG contacts with various organic and inorganic encapsulation layers. (a) 3D schematic of circular LIG electrode in contact with underlying graphene layer through various organic/inorganic encapsulation layers. (b) Extracted conductance of circular-type through-layer electrodes with varying thicknesses of PVC encapsulation compared to normal LIG without a PVC layer. (c) Optical images of circular-type through-layer LIG electrodes fabricated through a 60-μm-thick encapsulation layer (top) and *a* > 1 mm-thick encapsulation layer (bottom). The corresponding 2D schematics illustrate the differences in the conductance. Scale bar: 1 mm. Conductance of the through-layer LIG with (d) organic encapsulation layers (PVP, Na^+^ membrane, PAN, and TPU) and (e) inorganic encapsulation layers (Al_2_O_3_, SiO_2_, and HfO_2_). (f) *I*–*V* curves of the circular-type through-layer LIG electrode with a PVP encapsulation layer and normal LIG without encapsulation. The solid line represents the current flow in the pristine state after LIG formation, and the dotted line represents the current flow after O_2_ plasma treatment. All the experiments were conducted under the following laser parameters: power, 5%; scan rate, 15%. All error bars indicate standard deviation (*n* = 15).

With the optimized CO_2_ laser conditions, the effect of PVC thickness on through-layer LIG contact formation was further investigated. PVC-encapsulated graphene is irradiated by systematically varying the thickness of the PVC encapsulation layer. As the PVC layer thickness increased, a greater laser energy loss occurred during the etching process, leading to a wider gap between the through-layer LIG electrodes (Figure 3c). Consequently, the increased line resistance of the through-layer LIG led to a reduction in the overall conductance. Despite an increase in PVC thickness from 60 to 120 μm, electrical current continued to flow with only a slight reduction: 16.5% from 60 to 90 μm and 9.3% from 90 to 120 μm. However, when the PVC layer thickness exceeded 1 mm, the electrical current decreased significantly by approximately 90.86%, as most of the laser energy was absorbed during the etching of the thick PVC layer (Figure 3c).

Furthermore, the conductance of the PVC-encapsulated graphene was lower than that of graphene without encapsulation, confirming that a portion of the laser energy was consumed in etching the encapsulation layer before forming the through-layer LIG. However, by employing multiple CO_2_ laser-scribing cycles, a thicker encapsulation layer was successfully etched, enabling through-layer LIG contact formation with the graphene layer (Figure S2).

### Through-layer LIG Contacts for Various Organic/Inorganic Encapsulation layers

The through-layer LIG contact method can be applied to various organic and inorganic encapsulation layers, enabling its integration into diverse graphene-based devices. Organic encapsulation layers are typically used to functionalize graphene for chemical and biological sensing applications, enabling the conversion of target analytes into electrical signals.^18,52–55^ In contrast, inorganic encapsulation layers serve as gate dielectrics in graphene FETs and passivation layers to protect graphene from degradation and contamination.^30,32,56,57^

The conductance data presented in Figure 3d,e show that CO_2_ laser scribing successfully penetrates various organic (poly­(4-vinylphenol) (PVP), Na^+^ membrane, polyacrylonitrile (PAN), Thermoplastic Polyurethane (TPU), and Parylene C) and inorganic (Al_2_O_3_, SiO_2_, and HfO_2_) encapsulation layers, establishing a through-layer LIG contact with the underlying graphene. When PAN and TPU were used as the encapsulation layers, the conductance decreased compared to that observed with the PVP and Na^+^ membranes; however, a measurable current still flowed. The organic encapsulation materials were dissolved in different solvents at varying weight ratios to achieve suitable viscosities for bar coating, resulting in thickness variation. Furthermore, the differences in the chemical properties and heat resistance of the materials contributed to the variation in the conductance of the through-layer contact devices. All the raw *I*–*V* data and optical images of the organic and inorganic encapsulated through-layer contact devices are provided in the Supporting Information (Figures S3 and S4).

The encapsulation layer served as a protective barrier, preventing damage to the underlying graphene during O_2_ plasma exposure. O_2_ plasma etching is commonly used to pattern graphene and define channel regions by selectively removing graphene from designated areas. As shown in Figure 3f, the encapsulated through-layer LIG device exhibited minimal current reduction, indicating the effective protection of the graphene layer from O_2_ plasma during processing. In contrast, the unencapsulated device subjected to the same plasma treatment showed no measurable current, confirming that direct exposure to the O_2_ plasma severely degraded the graphene. The effects of the O_2_ plasma treatment on devices with other organic and inorganic encapsulation layers were further investigated and are presented in the Supporting Information (Figure S5).

Furthermore, in addition to the electrical characterization of the through-layer LIG devices with organic and inorganic encapsulation layers, the material composition and chemical structures of both the pristine encapsulation layers and through-layer LIG were analyzed using Fourier transform infrared spectroscopy (FT-IR), Raman spectroscopy, and X-ray photoelectron spectroscopy (XPS) (Figures S7–S9). Specifically, the pristine organic encapsulation layers and the formed LIG were analyzed using FT-IR. After CO_2_ laser ablation, the characteristic material peaks of hydroxyl and vinyl groups for PVP, vinyl groups for PVC, nitrile groups for PAN, and amine groups for TPU disappeared (Figure S7). Regardless of the encapsulation material, the LIG formed through these layers exhibited nearly identical transmittance profiles, showing C=C and C=O peaks within a broad spectral range of 1650 to 1740 cm^–1^. Additionally, Raman spectroscopy was conducted on LIG produced using various organic encapsulation layers. All the samples exhibited typical LIG features, including prominent D, G, and 2D bands (Figure S8). For the inorganic encapsulation layers, XPS analysis revealed that representative material peaks such as Al 2p, Si 2p, and Hf 4f disappeared after CO_2_ laser ablation. Instead, the appearance of the C 1s peak confirmed the formation of LIG (Figure S9).

### Through-Layer LIG for Multilayered Electrical Contacts

To demonstrate the multilayered electrical contacts, CVD-grown graphene was repeatedly transferred, with each graphene layer isolated by an encapsulation layer. After stacking the encapsulated graphene layers, CO_2_ laser scribing was performed to form the through-layer LIG contacts with each graphene layer. Figure 4a shows a schematic of this multilayer encapsulation/graphene stack, which was fabricated through successive cycles of graphene transfer and encapsulation-layer deposition. Notably, even as the number of stacked layers increased, the CO_2_ laser effectively etched through the encapsulation layers, allowing the LIG to grow and establish electrical contact with each graphene layer. In these experiments, Al_2_O_3_ and Parylene C were employed as encapsulation materials. For the Parylene C encapsulation layer, each graphene layer was transferred twice to prevent device degradation (Figure S10). The fabrication procedures are described in detail in Experimental Methods.

**Figure 4:**
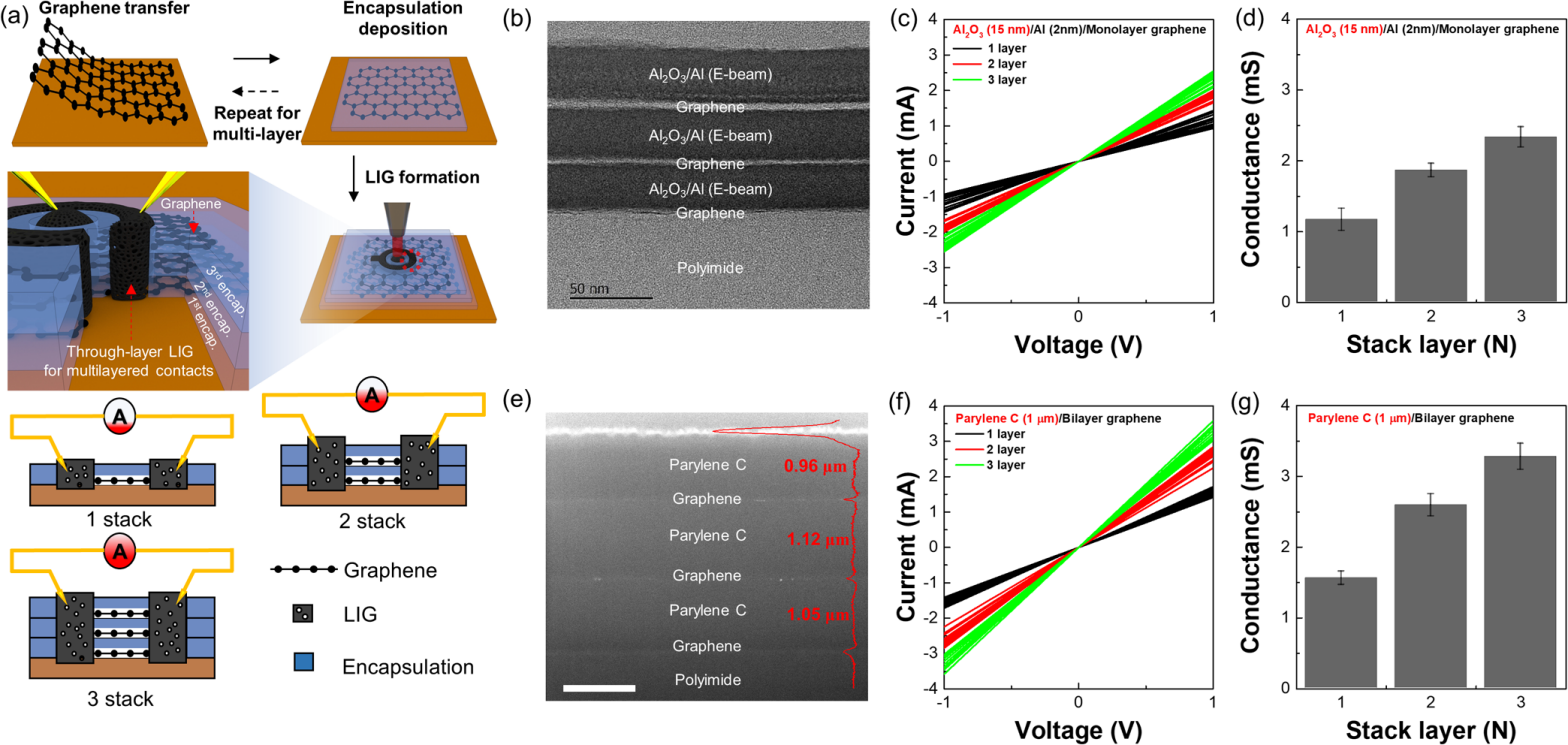
Through-layer LIG contacts for multilayer organic encapsulation/graphene and inorganic encapsulation/graphene stacks. (a) 3D schematic of the fabrication process for the multilayer encapsulation/graphene stack and through-layer LIG electrodes in contact with the graphene layer in each stack. (b) TEM image of multilayer Al_2_O_3_/Al/graphene stack on PI film. Scale bar: 50 nm. (c) *I*–*V* curves of circular-type LIG electrodes through multilayer Al_2_O_3_/Al/graphene stacks with one to three layers. (d) Extracted conductance values from the multilayer Al_2_O_3_/Al/graphene stacks. (e) SEM image of multilayer Parylene C/graphene stack on PI film. The red line, which represents the gray value profile of the SEM image for layer identification, is extracted using ImageJ. Scale bar: 1 μm. (f) *I*–*V* curves of circular-type LIG electrodes through multilayer Parylene C/graphene stacks with one to three layers. (g) Extracted conductance values from the multilayer Parylene C/graphene stacks. All experiments were conducted under the following laser parameters: power, 5%; scan rate, 15%. All error bars indicate standard deviation (*n* = 10).

The multilayer Al_2_O_3_/Al/graphene and Parylene C/graphene stacks were verified using transmission electron microscopy (TEM) and scanning electron microscopy (SEM) (Figure 4b,e). The TEM images clearly distinguished each Al_2_O_3_/Al encapsulation layer, confirming that each graphene layer is effectively isolated by these encapsulation layers. Similarly, the SEM images reveal that each Parylene C encapsulation layer is clearly distinguishable, with each graphene layer effectively isolated from the Parylene C layers. Additional high-magnification TEM images and energy-dispersive spectroscopy (EDS) mappings of the Al_2_O_3_/Al encapsulation stacks are provided in the Supporting Information (Figure S11).

Figure 4c,f show the *I*–*V* curves of the two-terminal circular-type through-layer LIG devices incorporating multiple Al_2_O_3_/Al/graphene and Parylene C/graphene encapsulation layers. The electrical current increased with the number of graphene/encapsulation layers. The conductance of the multilayer encapsulation/graphene stacks, extracted from these *I*–*V* curves, increases linearly with the number of stacked layers (Figure 4d,g). These results confirm the successful formation of multilayered electrical contacts, with each graphene layer effectively connected through the through-layer LIG structure.

Importantly, all the three graphene layers were successfully contacted using the through-layer LIG process. The first graphene layer was in direct contact with the PI film, which served as the precursor material for the LIG formation. In contrast, the second and third graphene layers were physically isolated from the PI film by the encapsulation layers but still exhibited reliable electrical contact via the through-layer LIG. Conventional multilayer electrical contacts typically require complex fabrication steps, including photolithography, RIE, metal deposition, and dual damascene processes. These methods involve multiple steps and expensive equipment as well as necessitate the use of additional etch-stop layers. In contrast, the proposed through-layer LIG contact method enables facile and scalable multilayer contact formation in a one-step CO_2_ laser-scribing process under ambient conditions.

### Top-Gate and Bottom-Gate Graphene Transistors with Through-layer LIG Contacts

In the first practical implementation of the through-layer LIG approach, top- and bottom-gate graphene transistors incorporating through-layer LIG source/drain (S/D) electrodes were fabricated using a CO_2_ laser-scribing process, replacing conventional rigid metal electrodes. Figure 5a,d show the schematics of the top- and bottom-gate graphene transistors, respectively. In these devices, the gate dielectric consisted of Al_2_O_3_ and the gate metal was composed of Ti/Au. The detailed fabrication procedures are provided in the Experimental Methods section. Briefly, gate electrode deposition, gate dielectric deposition, and graphene transfer were sequentially performed prior to CO_2_ laser scribing to form the through-layer LIG S/D electrodes. The gate stacks were verified by TEM and EDS mapping, in which all the component materials were clearly identified (Figure 5c,f).

**Figure 5:**
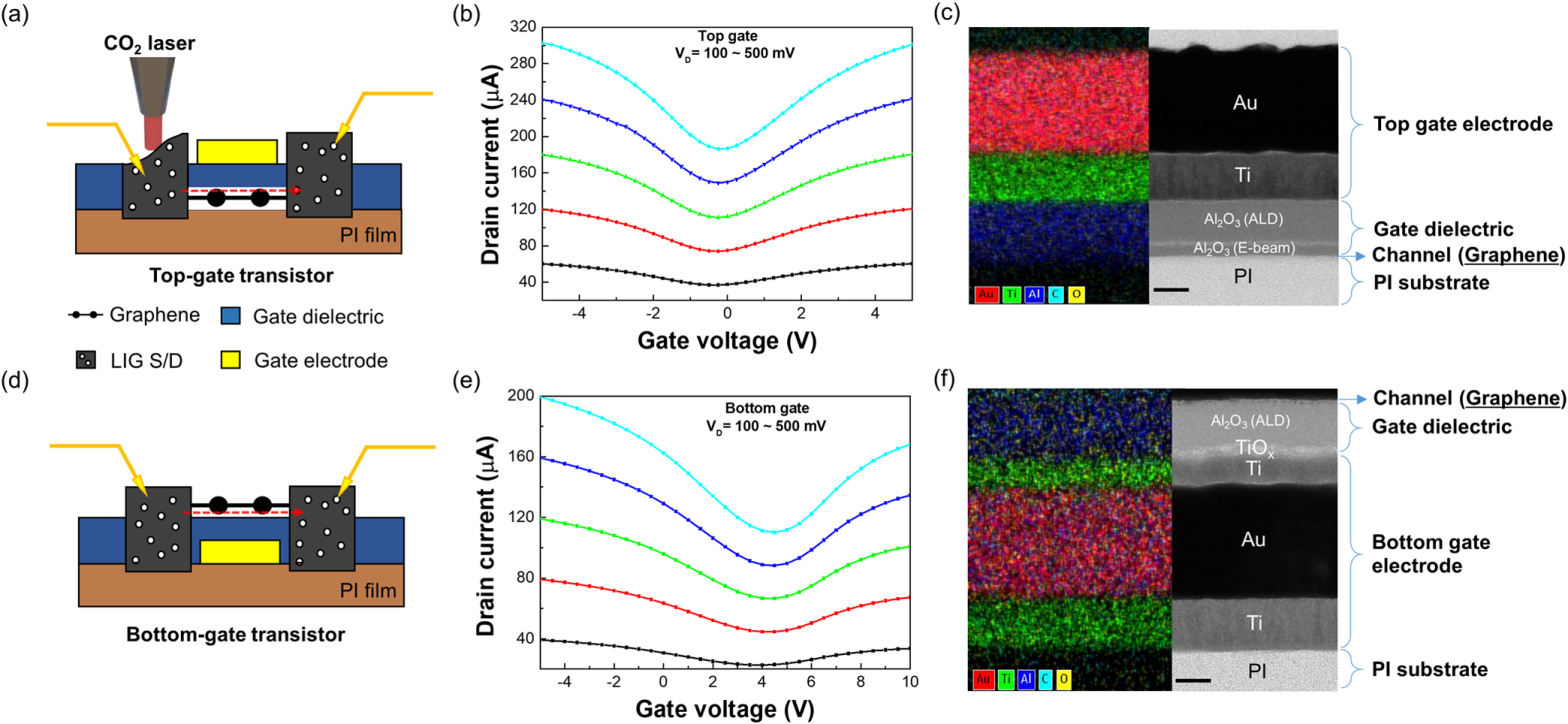
Graphene transistors with through-layer LIG source and drain electrodes. (a) Schematic, (b) transfer curves, and (c) cross-sectional TEM and EDS mapping images of top-gate graphene transistor incorporating through-layer LIG source/drain electrodes. (d) Schematic, (e) transfer curves, and (f) cross-sectional TEM and EDS mapping images of bottom-gate graphene transistor with through-layer LIG source/drain electrodes.

Graphene transistors with through-layer LIG S/D electrodes exhibit typical bipolar transfer characteristics, including a Dirac voltage corresponding to the minimum current (Figure 5b,e). Notably, the graphene transistors fabricated using the one-step CO_2_ laser-scribing process under ambient conditions demonstrated performances comparable to those of previously reported graphene transistors fabricated via conventional multistep processes.^36^ Table S1 (Supporting Information) compares key performance metrics with those of previously reported graphene transistors. The relatively modest mobility observed in our device can be attributed to several factors, including residue from the TRT transfer process, reduced gate control due to highly underlapped geometry,^58^ and carrier scattering from structural defects in the CVD-grown graphene.^59^ In the top-gate graphene transistor (Figure 5b), the drain current increases with increasing drain voltage, while an almost constant Dirac voltage is maintained. This behavior is a unique property of graphene, attributed to its Dirac cone-shaped band structure.^28,29^ In comparison, the bottom-gate device exhibited a positive shift in the Dirac voltage, possibly owing to oxygen adsorption on the graphene channel. This effect induces p-type doping, which can be attributed to the open-channel structure of the bottom-gate configuration.^60^

Notably, in the bottom-gate graphene transistor, the gate dielectric (i.e., Al_2_O_3_) was positioned between the graphene and PI film, and the through-layer LIG was formed in the final step to establish an electrical connection with the graphene (Figure 5e). This demonstrates that through-layer LIG contact with graphene is achievable even when the graphene is not in direct contact with the PI film.

### Through-Layer Laser Processing for Electrochemical Sensors

Without posttreatment, through-layer LIG devices offer a promising platform for biochemical sensors. In this configuration, the through-layer LIG in contact with graphene effectively functioned as an electrode to extract electrical signals from the target analytes, whereas the remaining encapsulation layer served as an efficient bio/chemical sensing interface. To demonstrate this sensing application, sodium ions were selected as model analytes because of their critical role in maintaining electrolyte balance and hydration levels. Monitoring sodium-ion concentration is particularly important in noninvasive sweat analysis to assess dehydration, electrolyte imbalances, and overall physiological health.^61,62^

To validate this approach, an electrochemical sodium-selective sensor was fabricated using the through-layer LIG contact method with a sodium-selective membrane layer. To achieve high selectivity toward Na^+^ ions, our sensor utilizes an ion-selective membrane incorporating a sodium-specific ionophore embedded in a plasticized polymer matrix. The selectivity stems from the preferential complexation of the ionophore with Na^+^, based on its cavity size and coordination chemistry, which minimize interference from competing ions such as K^+^ or Ca^2+^. This mechanism is well-described by the phase-boundary potential theory, which states that selective ion exchange at the membrane/sample interface governs the electrochemical response.^63^ Open-circuit potential (OCP) measurements were performed in solutions with varying sodium-ion concentrations, including both primary and interfering ions. In this sensor, a sodium-selective membrane-coated graphene was used as the working electrode. The through-layer LIG in contact with the graphene layer and the hole through both the sodium-selective membrane and PI film were simultaneously formed in the final step by CO_2_ laser scribing. Figure 6a shows a schematic of the sodium sensor, which features a through-layer LIG/graphene contact and a hole in the PI film, both of which were formed by CO_2_ laser scribing. The hole at the center of the sensor allows fluid collection when the device is attached to the skin, enabling the fluid to flow from the bottom (e.g., sweat) to the top (e.g., the sensor electrode). This fluid flow from beneath the PI film to the sensor surface was confirmed by optical images, showing green-dyed solutions passing through the microfluidic channels and holes (Figure 6i). In addition to enabling through-layer electrical contact, the creation of fluidic access holes is another advantage of through-layer laser processing. Detailed fabrication procedures are provided in the Experimental Methods section.

**Figure 6:**
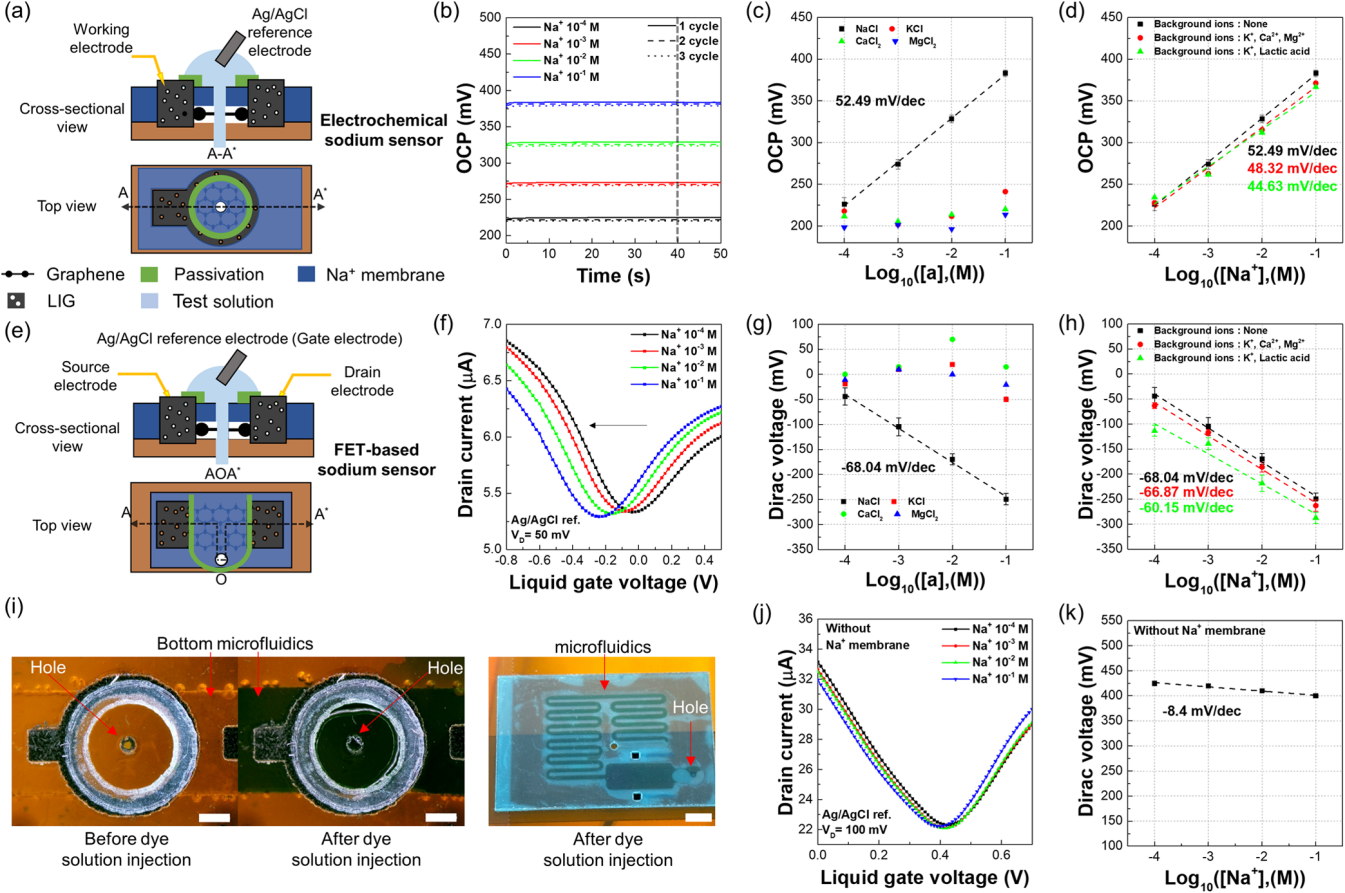
Electrochemical and electrical sodium-selective sodium sensors with through-layer LIG contacts. (a) 2D schematics of the electrochemical sodium sensor with through-layer LIG electrodes contacting the encapsulated graphene and a fluid-access hole. (b) OCP measurement for sodium-ion concentrations ranging from 0.1 to 100 mM. (c) Sensitivity and selectivity of the electrochemical sodium sensor extracted from the OCP data at 40 s, where various interfering ions (K^+^, Ca^2+^, and Mg^2+^) were used for the selectivity test. (d) Sensitivity of the electrochemical sodium sensor across sodium-ion concentrations of 0.1 to 100 mM, including various interfering background ions (K^+^, Ca^2+^, Mg^2+^, and lactic acid). (e) 2D schematic of the electrolyte-gated graphene FET-based sodium sensor featuring through-layer LIG electrodes in contact with encapsulated graphene and a fluid-access hole. (f) Transfer curve of the FET-based sodium sensor, measured for sodium-ion concentrations ranging from 0.1 to 100 mM. (g) Sensitivity and selectivity of electrical sodium sensor extracted from the Dirac voltage of the transfer curve. (h) Sensitivity of the electrical sodium sensor across sodium-ion concentrations from 0.1 to 100 mM, including various interfering background ions (K^+^, Ca^2+^, and Mg^2+^). (i) Optical images of Na sensors with fluid-access holes and attached microfluidics for the solution paths beneath the PI film to the sensor surface. Scale bar: 1 mm (left, middle), 4 mm (right) (j) Transfer curve of the electrolyte-gated graphene FET without a sodium-selective membrane, measured for sodium-ion concentrations ranging from 0.1 to 100 mM. (k) Sensitivity of the electrolyte-gated graphene FET without the sodium-selective membrane extracted from the Dirac voltage of the transfer curve.

As the sodium-ion concentration in the test solution increased, the OCP measured between the Ag/AgCl reference electrode and graphene working electrode also increased because of the increase in membrane potential (Figure 6b). The sodium sensor exhibited a sensitivity of 52.49 mV/dec, which was an acceptable value compared to the theoretical Nernstian limit,^61^ over a sodium-ion concentration range of 0.1 to 100 mM (Figure 6c). A detailed description of the sodium sensing mechanism is provided in the Supporting Information (Figure S12). Briefly, as the sodium-ion concentration in the test solution increased, the membrane potential also increased in the direction opposite to the sodium-ion concentration gradient between the membrane and test solution.

In addition to sodium ions, OCP measurements were performed for other interfering ions including potassium (K^+^), calcium (Ca^2+^), and magnesium (Mg^2+^) to evaluate the selectivity of the sensor. As shown in Figure 6c, the OCP exhibited negligible changes in response to varying concentrations of these interfering ions compared to the significant response observed for sodium ions. Selectivity coefficients of the sodium-selective membrane against K^+^, Ca^2+^, and Mg^2+^ were evaluated using the separate solution method;^64^ the corresponding results are summarized in Table S3 (Supporting Information). Furthermore, a sensitivity test was conducted using sodium ions in the presence of interfering background ions. As the total concentration of interfering ions increased (circle symbols: 1 mM each of K^+^, Ca^2+^, and Mg^2+^; triangle symbols: 1 mM K^+^ and 10 mM lactic acid), the activity of sodium ions decreased. This decrease in ion activity is considered the primary cause of sensor performance degradation, resulting in a reduction in sensitivity from 52.49 mV/dec to 48.32 and 44.63 mV/dec, respectively (Figure 6d). Based on these findings and consistent responses under background interference, the device is appropriately referred to as a sodium sensor.

An electrical sodium-selective sensor based on an FET configuration was also demonstrated. Figure 6e shows a schematic of the electrical Na sensor, which features a through-layer LIG/graphene contact and a hole in the PI film formed via CO_2_ laser scribing. The detailed fabrication procedure is described in Experimental Methods. The only difference between the electrochemical and electrical sodium sensors is the through-layer LIG electrode structure, as shown in Figure 6a,e. As the sodium-ion concentration increases, the transfer curve shifts to the left owing to a more positive membrane potential, where a gate voltage is applied using a conventional Ag/AgCl reference electrode. This sodium sensor exhibits a sensitivity of −68.04 mV/dec, which is above the Nernst limit and calculated from the extracted Dirac voltage, to sodium-ion concentration ranging from 0.1 to 100 mM (Figure 6g). The higher sensitivity beyond the Nernst limit could be attributed to the additive potential near the graphene defects.

To investigate the presence of additive potential contributing to sensitivity beyond the Nernst limit, transfer characteristics were measured in sodium-ion concentrations from 0.1 to 100 mM without a sodium-selective membrane. As the sodium-ion concentration increases, the transfer curve barely shifts to the left (Figure 6j). Based on the extracted Dirac voltage, a sensitivity of −8.4 mV/dec was calculated (Figure 6k). Considering the additive potential, the sensitivity of −68.04 mV/dec could be an acceptable value near theoretical Nernst limit. This approach is analogous to the demonstration of an additional boundary potential, arising from the interaction between sodium ions and SiO_2_ lattice, provided by Cao et al.^65^ To mitigate this positive boundary potential, they introduced a hydrogel layer between the sodium-selective membrane and the SiO_2_ surface. Consequently, the sensitivity decreased below the theoretical Nernst limit. The difference in sensitivity between the presence and absence of the hydrogel layer confirmed the existence of an additional positive boundary potential.

To evaluate whether through-layer LIG electrodes can serve as viable alternatives to conventional metal electrodes for Na sensing, Na sensors with Ti/Au (10/60 nm) electrodes were fabricated using electron-beam evaporation. The Ti/Au-based sodium sensor successfully detected changes in sodium-ion concentration and exhibited a sensitivity of −70 mV/dec (Figure S13), which is comparable to that of the LIG-based sodium sensor. These results confirm that through-layer LIG electrodes can effectively replace metal electrodes via a simple one-step CO_2_ laser-scribing process.

In addition, the transfer characteristics were measured at various concentrations of interfering ions (K^+^, Ca_,_^2+^ and Mg^2+^) for the selectivity test. As shown in Figure 6g, the Dirac voltage changed negligibly at various interfering ion concentrations ranging from 0.1 to 100 mM. A sensitivity test was conducted using the same method as that used for the electrochemical sodium sensor. As the total interfering ion concentrations increased, the activity of sodium ions decreased, resulting in a reduction in sensitivity from – 68.04 mV/dec to −66.87 and −60.15 mV/dec, respectively (Figure 6h).

## Conclusions

Herein, we present a simple and scalable one-step method for fabricating through-layer LIG electrical contacts with encapsulated graphene using CO_2_ laser scribing. This approach simultaneously etches the encapsulation layer and forms an LIG in direct contact with the underlying graphene, enabling reliable electrical contact through various organic and inorganic encapsulation materials. Real-time electrical measurements confirmed that through-layer LIG contacts were successfully formed at a sufficient laser energy during the single-step process. Furthermore, multilayered electrical contacts were demonstrated, with each graphene layer isolated by Al_2_O_3_ or Parylene C encapsulation layers. Notably, even when the graphene channel layer was physically separated from the PI film, which was the precursor for LIG formation, the through-layer LIG still established effective contact with each graphene layer. The versatility of through-layer LIG contacts has been demonstrated in various applications including electrical contacts with multilayer graphene stacks, top- and bottom-gate graphene transistors, and electrical and electrochemical sodium sensors. These devices exhibited characteristic electrical and sensing responses depending on the structure and configuration. The solid-state graphene FETs, with Al_2_O_3_ gate dielectrics, showed ambipolar transport and reliable gate modulation, whereas the ion-sensitive FETs demonstrated Na^+^-dependent signal modulation via ion-selective membranes. In addition, electrochemical Na^+^ sensors utilizing graphene working electrodes exhibited a distinct Nernstian response. These results confirm the versatility of our platform for diverse graphene-based electronic and chemical sensing applications. In contrast to conventional multistep fabrication methods involving photolithography, etching, and metal deposition, the proposed technique streamlines the process into a single step and operates entirely under ambient conditions, eliminating the need for vacuum equipment or cleanroom infrastructure. Overall, this paper presents a promising platform for the low-cost and scalable fabrication of encapsulated graphene devices, particularly for application in flexible electronics, FETs, and chemical sensors.

## Experimental Methods

### Materials

PVP, PVC, PAN, TPU, poly­(melamine-*co*-formaldehyde) methylated (PMF), propylene glycol monomethyl ether acetate (PGMEA), *N*,*N*-dimethylformamide (DMF), methyl ethyl ketone (MEK), ammonium persulfate (APS), tetrahydrofuran (THF), sodium ionophore X, and bis­(2-ethylhexyl) adipate (DOA) were purchased from Sigma-Aldrich (St. Louis, MO). Poly­(methyl methacrylate) (PMMA) was purchased from Kayaku Advanced Materials, Inc. (Westborough, MA). TRT was purchased from Jinsung Chemical Co. Ltd. (Icheon, South Korea). A 125-μm-thick PI film (Kapton HN grade) was purchased from DuPont (Wilmington, DE). Polyethylene terephthalate (PET) films were purchased from FilmBank (Siheung, South Korea). CVD-grown monolayer graphene on Cu foil (thickness: 11 μm) was purchased from Chamgraphene Co., Ltd. (Suwon, South Korea). Silicone elastomer (Kwik-Cast) was purchased from World Precision Instruments, Inc. (Sarasota, FL). A polydimethylsiloxane (PDMS) thin film (200 μm) was purchased from CSCRIE Corporation (Kyoto, Japan).

### Real-Time In Situ Through-Layer LIG/Graphene Contact Measurement

First, to transfer bilayer graphene onto a PI film, CVD-grown graphene on Cu foil was flattened using a laminator and then adhered to a TRT. To remove the unwanted backside graphene, O_2_ plasma treatment was performed (100 W, 30 sccm, 50 mTorr, 5 min). The copper layer was subsequently etched using 0.1 M APS for 1 h, followed by rinsing with deionized (DI) water and drying with N_2_ gas. The graphene/TRT film was laminated onto the PI substrate using a laminator and heated on a hot plate (100 °C for 10 s). This process facilitated the successful transfer of graphene onto the PI film, as the TRT lost its adhesive properties upon heating.

A graphene channel with a width of 2 mm was defined by O_2_ plasma etching (100 W, 30 sccm, 50 mTorr, 5 min) using a PDMS thin film as an etching mask. Subsequently, a PVC solution was prepared for encapsulation. PVC and DOA were dissolved in THF in a weight ratio of 1:2:2, with DOA serving as a plasticizer to enhance PVC flexibility. The prepared PVC solution was applied to the graphene layer using a bar coater to achieve a uniform film thickness of 500 μm and was then dried under ambient conditions overnight.

For the real-time electrical current measurements, one pair of interdigitated through-layer LIG electrodes was formed using CO_2_ laser scribing (power: 5%, scan rate: 13%). Another pair of interdigitated through-layer LIG electrodes was fabricated under the same CO_2_ laser-scribing conditions, except for the electrode fingers. Both ends of the interdigitated electrodes were connected to a source meter using a silver paste and carbon tape. Finally, *in situ* electrical measurements were performed using a CO_2_ laser for direct writing to form three-finger through-layer LIG electrodes in contact with the encapsulated graphene layer.

### Fabrication of Organic and Inorganic Encapsulation Layers on Graphene

First, to transfer monolayer graphene onto a PI film, CVD-grown graphene on a Cu foil was flattened using a laminator, followed by adhesion to the TRT. To remove the unwanted backside graphene, an O_2_ plasma treatment was performed (100 W, 30 sccm, 50 mTorr, 5 min). The copper layer was subsequently etched using 0.1 M APS for 1 h, followed by rinsing with deionized (DI) water and drying with N_2_ gas. The graphene/TRT film was laminated onto the PI substrate using a laminator and heated on a hot plate (100 °C for 10 s). Graphene was successfully transferred to the PI film as the TRT lost its adhesive properties when heated.

To encapsulate the graphene layer with organic materials, various organic solutions have been prepared and coated using bar coating or an applicator with a controlled thickness. PVP and PMF were dissolved in PGMEA at a weight ratio of 1:5:3, with PMF serving as a cross-linker. PAN and TPU were dissolved in MEK/DMF in weight ratios of 20:18:62 and 15:25:60, respectively. The prepared organic solutions were applied to the graphene layer by bar coating to achieve a uniform film thickness of 500 μm for PVP, Na^+^ membrane, PAN, and TPU, and were subsequently dried under ambient conditions overnight. Specifically, PVP-encapsulated graphene was cross-linked by heating on a hot plate (180 °C for 1 h).

To investigate the effect of PVC thickness on through-layer LIG contact formation, graphene layers were encapsulated with PVC using an applicator to achieve thicknesses of 60, 90, and 120 μm, followed by drying under ambient conditions overnight. Except for these specific thicknesses, all the organic encapsulation layers were deposited using the bar-coating method.

In addition to organic materials, graphene layers were encapsulated with inorganic materials using atomic layer deposition (ALD) or plasma-enhanced ALD (PEALD). Prior to ALD, a 5 nm-thick Al_2_O_3_ layer was deposited on the graphene surface via electron-beam evaporation to serve as a seed layer. This was followed by O_2_ plasma treatment (100 W, 30 sccm, 50 mTorr, 5 min) to prevent the exposure of graphene to the ALD chamber. Subsequently, 15 nm-thick layers of Al_2_O_3_, SiO_2_, and HfO_2_ were deposited over the graphene layer via ALD or PEALD using the following precursors: trimethylaluminum (TMA), di­(isopropylamino)­silane (DIPAS), tetrakis­(ethylmethylamido)hafnium (TEMAHf), and H_2_O. Finally, the encapsulated graphene was irradiated with a CO_2_ laser at 5% power and a 15% scan rate for through-layer LIG contact formation. A circular LIG electrode was used to measure the electrical current between the LIG and graphene layer without applying a graphene patterning process.

### CO_2_ Laser Irradiation for Through-Layer LIG Contacts

A computer-controlled CO_2_ laser (Universal Laser Systems VLS 2.30DT) with a wavelength of 10.6 μm, a maximum power of 30 W (100%), and a maximum scan rate of 10 in/s (100%, vector mode) was used to form through-layer LIG contacts via laser scribing on an encapsulated graphene/PI film. The through-layer LIG was formed using a direct writing method in which the pattern was imported from a CAD file. The laser system allowed for modifications in the power and scan rate settings. The same pulse per inch value of 1000 was used in all experiments. All laser-scribing processes were performed under ambient conditions.

### Fabrication of Multilayer Graphene/Organic and Inorganic Encapsulation Stacks

Graphene was transferred onto the PI film using the conventional PMMA transfer method. First, PMMA was spin-coated onto CVD-grown graphene on a Cu foil. To remove the unwanted backside graphene, O_2_ plasma treatment was performed (100 W, 30 sccm, 50 mTorr, 5 min). The underlying copper layer was etched in a 0.1 M APS solution for 1 h, followed by rinsing with DI water. The resulting graphene/PMMA film was scooped onto the PI substrate and dried overnight under ambient conditions. The PMMA was subsequently removed by immersion in acetone. After graphene transfer, a 1-μm-thick Parylene C encapsulation layer was deposited using a Parylene coater with dipara-xylylene as the precursor.

For the stacks with inorganic encapsulation layers, graphene was transferred onto the PI film using the TRT transfer method. Following the transfer, a 15 nm/2 nm-thick Al_2_O_3_/Al encapsulation layer was deposited by electron-beam evaporation.

To fabricate multilayer graphene/encapsulation stacks, the graphene transfer and encapsulation processes were sequentially repeated. Finally, the stacked, encapsulated graphene structures were irradiated with a CO_2_ laser at 5% power and a 15% scan rate to establish through-layer LIG contacts. A circular LIG electrode was used to measure the electrical current between the LIG and graphene layer without graphene patterning.

### Fabrication of Top-Gate and Bottom-Gate Graphene Transistors

To fabricate top-gate graphene transistors, graphene was transferred onto a PI film using the TRT transfer method. To define the graphene channel, O_2_ plasma treatment (100 W, 30 sccm, 50 mTorr, 5 min) was performed to etch the unwanted regions of the graphene using a PDMS layer as a mask. A 5 nm-thick Al_2_O_3_ layer was then deposited over the graphene by electron-beam evaporation as a seed layer for ALD, followed by an additional O_2_ plasma treatment (100 W, 30 sccm, 50 mTorr, 5 min) to prevent direct exposure of the graphene to the ALD chamber. Subsequently, a 15 nm-thick Al_2_O_3_ gate dielectric layer was deposited via ALD, using TMA and H_2_O as precursors. A 10/60 nm-thick Ti/Au gate electrode was deposited via electron-beam evaporation through a PET mask fabricated via CO_2_ laser processing.

To fabricate bottom-gate graphene transistors, graphene was transferred onto a PI film with a predeposited 10/50/5 nm-thick Ti/Au/Ti gate electrode using the PMMA transfer method. Similar to the top-gate transistor fabrication, O_2_ plasma treatment was performed to define the graphene channel, following the ALD deposition of a 15 nm-thick Al_2_O_3_ gate dielectric layer.

Finally, through-layer LIG source/drain (S/D) electrodes were fabricated using CO_2_ laser scribing (5% power and 15% scan rate). It should be noted that all the through-layer LIG S/D electrodes were fabricated in the final step, even though the gate dielectric was already deposited on the PI film.

### Synthesis of Sodium-Selective Membrane

A sodium-selective membrane cocktail was prepared by dissolving sodium ionophore X (1 wt %), PVC (33 wt %), and DOA (66 wt %) in THF. A total of 100 mg of the membrane cocktail mixture was dissolved in 500 μL of THF. To prevent solvent evaporation and maintain viscosity, the prepared membrane cocktail was stored at 4 °C prior to use.

### Fabrication of Sodium-Selective Sensors

Graphene was transferred onto the PI film using the TRT transfer method to fabricate an electrochemical sodium-selective sensor. To define the graphene working electrode region, O_2_ plasma treatment (100 W, 30 sccm, 50 mTorr, 5 min) was performed to etch unwanted graphene using the TRT layer as a mask. To functionalize the active graphene electrode as a Na-selective electrode, a Na-selective membrane was coated onto the graphene surface using a bar coater and dried overnight under ambient conditions. Finally, a through-layer LIG electrode, which makes contact with the graphene layer, and a hole that allows the solution to pass from the bottom to the top of the substrate, were fabricated by CO_2_ laser scribing (5% power, 15% scan rate).

In addition to an electrochemical sodium-selective sensor, an electrical sodium-selective sensor operated using an electrolyte-gated FET was fabricated. The fabrication processes for both the electrical and electrochemical sensors were identical, except for differences in the graphene channel pattern and shape of the through-layer LIG electrodes.

Finally, a solution reservoir made of either a Kwik-cast silicone elastomer or a patterned TRT layer was attached to the device.

### Preparation of Test Solutions and Artificial Sweat for Sodium Sensing

To evaluate the sensitivity and selectivity of the sodium-selective sensors, NaCl, KCl, CaCl_2_, and MgCl_2_ solutions were prepared with concentrations ranging from 0.1 to 100 mM. To validate the effect of ionic strength on sensor sensitivity, NaCl solutions (0.1–100 mM) containing 1 mM KCl, CaCl_2_, and MgCl_2_ as background interfering ions were also prepared.

An artificial sweat solution was prepared by dissolving NaCl (0.1–100 mM), KCl (1 mM), and lactic acid (10 mM) in deionized (DI) water. All the primary ions, interfering ions, and organic acids were dissolved in DI water.

### Electrical and Electrochemical Measurements

Electrical measurements were conducted using a SourceMeter (Model 2612 B, Keithley Instruments, LLC) to measure the *I*–*V* characteristics of the circular two-terminal devices as well as the transfer characteristics of the top-gate and bottom-gate graphene transistors and electrolyte-gated graphene transistors for sodium sensing.

For the electrolyte-gated graphene transistor, an Ag/AgCl reference electrode (R0302, The Solution, South Korea) was used to apply the gate voltage. The drain current was measured under a constant drain voltage of 50 mV, while the gate voltage applied to the reference electrode was swept from – 0.8 to 0.5 V. The source electrode was ground electrically.

The OCP measurements were performed using an electrochemical workstation (CHI760E, CH Instruments, Inc.). In this setup, the Ag/AgCl reference electrode was connected to the reference terminal of the electrochemical workstation, and the through-layer LIG electrode was connected to the working-electrode terminal. In OCP measurements, the reference terminal exhibited a high input impedance, and the system recorded the equilibrium potential between the Ag/AgCl reference electrode and working-electrode surface exposed to the electrolyte, in the absence of any external bias.

## Supplementary Material







## Data Availability

Data supporting the findings of this study are available from the corresponding author upon reasonable request.

## Notes

Additional experimental details, methods, and results; CO_2_ laser average fluence calculation; optimization of through-layer LIG formation through the PVC encapsulation layer; CO_2_ laser multiscribing for etching thick encapsulation layers; raw *I*–*V* data for organic and inorganic encapsulated through-layer contact devices; optical top-view images of organic and inorganic encapsulated through-layer contact devices; effects of O_2_ plasma treatment on the encapsulated graphene layer; encapsulation crack adjacent to the through-layer LIG; Fourier transform infrared spectroscopy (FT-IR) of various organic encapsulation layers and through-layer LIG; Raman spectroscopy of through-layer LIG with various organic encapsulation layers; X-ray photoelectron spectroscopy (XPS) of various inorganic encapsulation layers and through-layer LIG; graphene transfer for multilayered electrical contacts based on Parylene C encapsulation layer; transmission electron microscopy (TEM) images and energy-dispersive spectroscopy (EDS) mapping of Al_2_O_3_/Al encapsulation stacks; Na sensing mechanism using a Na-selective membrane; electrical Na sensors based on conventional metal contacts; comparison of the performance of the graphene transistor with that of previously reported devices; comparison of the performance of the sodium sensor with that of previously reported ion-selective electrodes and ion-sensitive field-effect transistors; Selectivity coefficients of the sodium-selective membrane (PDF)
Real-time electrical monitoring of through-layer LIG contact formation in the encapsulated graphene under sufficient laser energy (MP4)
Real-time electrical monitoring of through-layer LIG contact formation in the encapsulated graphene under insufficient and sufficient laser energies (MP4)

## References

[1] Lin Y. M., Dimitrakopoulos C., Jenkins K. A., Farmer D. B., Chiu H. Y., Grill A., Avouris P. (2010). 100-GHz Transistors from Wafer-Scale
Epitaxial Graphene. Science.

[2] Novoselov K. S. (2016). Electric Field Effect
in Atomically Thin Carbon Films. Science.

[3] Cao M., Xiong D. B., Yang L., Li S., Xie Y., Guo Q., Li Z., Adams H., Gu J., Fan T., Zhang X., Zhang D. (2019). Ultrahigh Electrical
Conductivity
of Graphene Embedded in Metals. Adv. Funct.
Mater..

[4] Vadukumpully S., Paul J., Mahanta N., Valiyaveettil S. (2011). Flexible Conductive
Graphene/Poly­(Vinyl Chloride) Composite Thin Films with High Mechanical
Strength and Thermal Stability. Carbon.

[5] Lee C., Wei X., Kysar J. W., Hone J. (2008). Measurement of the Elastic Properties
and Intrinsic Strength of Monolayer Graphene. Science.

[6] Nair R. R., Blake P., Grigorenko A. N., Novoselov K. S., Booth T. J., Stauber T., Peres N. M. R., Geim A. K. (2008). Fine Structure
Constant Defines Visual Transparency of Graphene. Science.

[7] Liu N., Chortos A., Lei T., Jin L., Kim T. R., Bae W. G., Zhu C., Wang S., Pfattner R., Chen X., Sinclair R., Bao Z. (2017). Ultratransparent and
Stretchable Graphene Electrodes. Sci. Adv..

[8] Miao J., Liu H., Li Y., Zhang X. (2018). Biodegradable Transparent Substrate
Based on Edible Starch-Chitosan Embedded with Nature-Inspired Three-Dimensionally
Interconnected Conductive Nanocomposites for Wearable Green Electronics. ACS Appl. Mater. Interfaces.

[9] Amontree J., Yan X., DiMarco C. S., Levesque P. L., Adel T., Pack J., Holbrook M., Cupo C., Wang Z., Sun D., Biacchi A. J., Wilson-Stokes C. E., Watanabe K., Taniguchi T., Dean C. R., Hight Walker A. R., Barmak K., Martel R., Hone J. (2024). Reproducible Graphene Synthesis by Oxygen-Free Chemical Vapour Deposition. Nature.

[10] Mattevi C., Kim H., Chhowalla M. (2011). A Review of
Chemical Vapour Deposition of Graphene
on Copper. J. Mater. Chem..

[11] De
Fazio D., Purdie D. G., Ott A. K., Braeuninger-Weimer P., Khodkov T., Goossens S., Taniguchi T., Watanabe K., Livreri P., Koppens F. H. L., Hofmann S., Goykhman I., Ferrari A. C., Lombardo A. (2019). High-Mobility, Wet-Transferred
Graphene Grown by Chemical Vapor Deposition. ACS Nano.

[12] Kim S., Nah J., Jo I., Shahrjerdi D., Colombo L., Yao Z., Tutuc E., Banerjee S. K. (2009). Realization of a High Mobility Dual-Gated
Graphene Field-Effect Transistor with Al_2_O_3_ Dielectric. Appl. Phys. Lett..

[13] Meric I., Han M. Y., Young A. F., Ozyilmaz B., Kim P., Shepard K. L. (2008). Current Saturation
in Zero-Bandgap, Top-Gated Graphene
Field-Effect Transistors. Nat. Nanotechnol..

[14] Pandey A., Gurbuz Y., Ozguz V., Niazi J. H., Qureshi A. (2017). Graphene-Interfaced
Electrical Biosensor for Label-Free and Sensitive Detection of Foodborne
Pathogenic E. Coli O157:H7. Biosens. Bioelectron..

[15] Dharuman V., Hahn J. H., Jayakumar K., Teng W. (2013). Electrochemically Reduced
Graphene-Gold Nano Particle Composite on Indium Tin Oxide for Label
Free Immuno Sensing of Estradiol. Electrochim.
Acta.

[16] Elshafey R., Siaj M., Tavares A. C. (2016). Au Nanoparticle Decorated Graphene
Nanosheets for Electrochemical Immunosensing of P53 Antibodies for
Cancer Prognosis. Analyst.

[17] Fowler J. D., Allen M. J., Tung V. C., Yang Y., Kaner R. B., Weiller B. H. (2009). Practical Chemical
Sensors from Chemically Derived
Graphene. ACS Nano.

[18] Xue M., Mackin C., Weng W. H., Zhu J., Luo Y., Luo S. X. L., Lu A. Y., Hempel M., McVay E., Kong J., Palacios T. (2022). Integrated Biosensor
Platform Based
on Graphene Transistor Arrays for Real-Time High-Accuracy Ion Sensing. Nat. Commun..

[19] Lee S. K., Humayun
Kabir S. M., Sharma B. K., Kim B. J., Cho J. H., Ahn J. H. (2014). Photo-Patternable Ion Gel-Gated Graphene Transistors
and Inverters on Plastic. Nanotechnology.

[20] Khan U., Kim T. H., Ryu H., Seung W., Kim S. W. (2017). Graphene
Tribotronics for Electronic Skin and Touch Screen Applications. Adv. Mater..

[21] Eda G., Fanchini G., Chhowalla M. (2008). Large-Area
Ultrathin Films of Reduced
Graphene Oxide as a Transparent and Flexible Electronic Material. Nat. Nanotechnol..

[22] Kang J., Hwang S., Kim J. H., Kim M. H., Ryu J., Seo S. J., Hong B. H., Kim M. K., Choi J. B. (2012). Efficient
Transfer of Large-Area Graphene Films onto Rigid Substrates by Hot
Pressing. ACS Nano.

[23] Kim H. H., Lee S. K., Lee S. G., Lee E., Cho K. (2016). Wetting-Assisted
Crack- and Wrinkle-Free Transfer of Wafer-Scale Graphene onto Arbitrary
Substrates over a Wide Range of Surface Energies. Adv. Funct. Mater..

[24] Chen X. D., Liu Z. B., Zheng C. Y., Xing F., Yan X. Q., Chen Y., Tian J. G. (2013). High-Quality
and Efficient Transfer
of Large-Area Graphene Films onto Different Substrates. Carbon.

[25] Zhang G., Güell A. G., Kirkman P. M., Lazenby R. A., Miller T. S., Unwin P. R. (2016). Versatile Polymer-Free Graphene Transfer
Method and
Applications. ACS Appl. Mater. Interfaces.

[26] Deng B., Hsu P. C., Chen G., Chandrashekar B. N., Liao L., Ayitimuda Z., Wu J., Guo Y., Lin L., Zhou Y., Aisijiang M., Xie Q., Cui Y., Liu Z., Peng H. (2015). Roll-to-Roll Encapsulation of Metal Nanowires between
Graphene and Plastic Substrate for High-Performance Flexible Transparent
Electrodes. Nano Lett..

[27] Bae S., Kim H., Lee Y., Xu X., Park J. S., Zheng Y., Balakrishnan J., Lei T., Ri Kim H., Song Y. Il., Kim Y. J., Kim K. S., Özyilmaz B., Ahn J. H., Hong B. H., Iijima S. (2010). Roll-to-Roll
Production
of 30-Inch Graphene Films for Transparent Electrodes. Nat. Nanotechnol..

[28] Liu H., Liu Y., Zhu D. (2011). Chemical Doping
of Graphene. J. Mater. Chem..

[29] Partoens B., Peeters F. M. (2006). From Graphene to
Graphite: Electronic Structure around
the K Point. Phys. Rev. B.

[30] Hsieh Y., Hofmann M., Chang K., Jhu J. G., Li Y., Chen K. Y. (2013). Complete Corrosion
Inhibition through Graphene Defect
Passivation. ACS Nano.

[31] Wang L., Zihlmann S., Baumgartner A., Overbeck J., Watanabe K., Taniguchi T., Makk P., Schönenberger C. (2019). In Situ Strain
Tuning in hBN-Encapsulated Graphene Electronic Devices. Nano Lett..

[32] Alexandrou K., Petrone N., Hone J., Kymissis I. (2015). Encapsulated Graphene
Field-Effect Transistors for Air Stable Operation. Appl. Phys. Lett..

[33] Seo Y., Masubuchi S., Watanabe E., Onodera M., Moriya R., Watanabe K., Taniguchi T., Machida T. (2020). Selective Etching of
Hexagonal Boron Nitride by High-Pressure CF4 Plasma for Individual
One-Dimensional Ohmic Contacts to Graphene Layers. Appl. Phys. Lett..

[34] Kretinin A. V., Cao Y., Tu J. S., Yu G. L., Jalil R., Novoselov K. S., Haigh S. J., Gholinia A., Mishchenko A., Lozada M., Georgiou T., Woods C. R., Withers F., Blake P., Eda G., Wirsig A., Hucho C., Watanabe K., Taniguchi T., Geim A. K., Gorbachev R. V. (2014). Electronic
Properties of Graphene Encapsulated with Different Two-Dimensional
Atomic Crystals. Nano Lett..

[35] Athas J., Ereifej J., Torres Quiñones J., Abrams A., Yun M. (2024). A Scalable Top-Gate Graphene Field
Effect Transistor with a Polydimethylsiloxane
Dielectric. Nano Trends.

[36] Nayfeh O. M. (2011). Graphene
Transistors on Mechanically Flexible Polyimide Incorporating Atomic-Layer-Deposited
Gate Dielectric. IEEE Electron Device Lett..

[37] Smith J. T., Franklin A. D., Farmer D. B., Dimitrakopoulos C. D. (2013). Reducing
Contact Resistance in Graphene Devices through Contact Area Patterning. ACS Nano.

[38] Telford E. J., Benyamini A., Rhodes D., Wang D., Jung Y., Zangiabadi A., Watanabe K., Taniguchi T., Jia S., Barmak K., Pasupathy A. N., Dean C. R., Hone J. (2018). Via Method
for Lithography Free Contact and Preservation of 2D Materials. Nano Lett..

[39] Wang L., Meric I., Huang P. Y., Gao Q., Gao Y., Tran H., Taniguchi T., Watanabe K., Campos L. M., Muller D. A., Guo J., Kim P., Hone J., Shepard K. L., Dean C. R. (2013). One-Dimensional
Electrical Contact
to a Two-Dimensional Material. Science.

[40] Lee H. Y., Wang Z., Chen G., Holtzman L. N., Yan X., Amontree J., Zangiabadi A., Watanabe K., Taniguchi T., Barmak K., Kim P., Hone J. C. (2024). In Situ via Contact
to hBN-Encapsulated Air-Sensitive Atomically Thin Semiconductors. ACS Nano.

[41] Kim M., Mackenzie D. M. A., Kim W., Isakov K., Lipsanen H. (2021). All-Parylene
Flexible Wafer-Scale Graphene Thin Film Transistor. Appl. Surf. Sci..

[42] Deepak, J. R. ; Anirudh, R. P. ; Saran Sundar, S. Applications of Lasers in Industries and Laser Welding: A Review Mater. Today: Proc. 2023, 10.1016/j.matpr.2023.02.102.

[43] Stournaras A., Stavropoulos P., Salonitis K., Chryssolouris G. (2009). An Investigation
of Quality in CO_2_ Laser Cutting of Aluminum. CIRP J. Manuf. Sci. Technol..

[44] Lin J., Peng Z., Liu Y., Ruiz-Zepeda F., Ye R., Samuel E. L. G., Yacaman M. J., Yakobson B. I., Tour J. M. (2014). Laser-Induced
Porous Graphene Films from Commercial Polymers. Nat. Commun..

[45] Ma X., Wang S., Fu Z., Gao H., Shao Y., Li X., Yan X. (2025). Laser-Induced Porous
Graphene-Based Materials and Its
Potential Application in Lithium – Ion Batteries. ACS Omgea.

[46] Dallinger A., Keller K., Fitzek H., Greco F. (2020). Stretchable and Skin-Conformable
Conductors Based on Polyurethane/Laser-Induced Graphene. ACS Appl. Mater. Interfaces.

[47] Jin
Na Y., Park J., Park S. C., Gyun Park W., Kim K. W., Wang B., Ahn J. H. (2024). Laser-Induced Graphene
as Versatile Sensing Electrodes for Extended-Gate Field-Effect Transistors. IEEE Sens. J..

[48] Ren G., Fan H., Zhang L., He S., Zhu C., Gao K., Zhang Y., Wang J., Kang X., Song Y., Gong Z., Li G., Lu G., Yu H. D. (2023). A Laser-Induced
Graphene-Based Flexible and All-Carbon Organic Electrochemical Transistor. J. Mater. Chem. C.

[49] Abdulhafez M., Tomaraei G. N., Bedewy M. (2021). Fluence-Dependent
Morphological Transitions
in Laser-Induced Graphene Electrodes on Polyimide Substrates for Flexible
Devices. ACS Appl. Nano Mater..

[50] Cheng L., Yeung C. S., Huang L., Ye G., Yan J., Li W., Yiu C., Chen F. R., Shen H., Tang B. Z., Ren Y., Yu X., Ye R. (2024). Flash Healing of Laser-Induced Graphene. Nat.
Commun..

[51] Park W. G., Park S. C., Cho H. J., Oh Y. W., Kang I. S., Ahn J. H. (2024). In Situ Electrical
Contacts to Graphene by Laser Scribing. ACS
Appl. Nano Mater..

[52] von
Lüders L., Tilmann R., Lee K., Bartlam C., Stimpel-Lindner T., Nevanen T. K., Iljin K., Knirsch K. C., Hirsch A., Duesberg G. S. (2023). Functionalisation of Graphene Sensor
Surfaces for the Specific Detection of Biomarkers. Angew. Chem..

[53] Dou C., Wu Z., Chen W., Yan H., Li D., You X. Q., Chen Y. S., Zhou C., Chen S., Zhuang P., Liu J. (2024). Au-Functionalized Wrinkle Graphene Biosensor for Ultrasensitive Detection
of Interleukin-6. Carbon.

[54] Kong D., Wang X., Gu C., Guo M., Wang Y., Ai Z., Zhang S., Chen Y., Liu W., Wu Y., Dai C., Guo Q., Qu D., Zhu Z., Xie Y., Liu Y., Wei D. (2021). Direct SARS-CoV-2 Nucleic
Acid Detection by Y-Shaped
DNA Dual-Probe Transistor Assay. J. Am. Chem.
Soc..

[55] Gao J., Wang C., Wang C., Chu Y., Wang S., Sun M. Y., Ji H., Gao Y., Wang Y., Han Y., Song F., Liu H., Zhang Y., Han L. (2022). Poly-L-Lysine-Modified
Graphene Field-Effect Transistor Biosensors for Ultrasensitive Breast
Cancer MiRNAs and SARS-CoV-2 RNA Detection. Anal. Chem..

[56] Simionescu O. G., Avram A., Adiaconita B., Preda P., Pârvulescu C., Năstase F., Chiriac E., Avram M. (2023). Field-Effect Transistors
Based on Single-Layer Graphene and Graphene-Derived Materials. Micromachines.

[57] Generalov A. A., Viisanen K. L., Senior J., Ferreira B. R., Ma J., Möttönen M., Prunnila M., Bohuslavskyi H. (2024). Wafer-Scale
CMOS-Compatible Graphene Josephson Field-Effect Transistors. Appl. Phys. Lett..

[58] Ahmad M. A., Kumar J. (2023). The Understanding of
the Impact of Efficiently Optimized Underlap
Length on Analog/RF Performance Parameters of GNR-FETs. Sci. Rep..

[59] Ballestas K., Diego Zapata J., Ramírez D. (2023). Quantification of Coverage, Uniformity
and Residues for CVD Monolayer Graphene Transfer Process Based on
Image Analysis. Appl. Surf. Sci..

[60] Aguirre C. M., Levesque P. L., Paillet M., Lapointe F., St-Antoine B. C., Desjardins P., Martel R. (2009). The Role of the Oxygen/Water Redox
Couple in Suppressing Electron Conduction in Field-Effect Transistors. Adv. Mater..

[61] Rovira M., Lafaye C., Wang S., Fernandez-Sanchez C., Saubade M., Liu S. C., Jimenez-Jorquera C. (2023). Analytical
Assessment of Sodium ISFET Based Sensors for Sweat Analysis. Sens. Actuators, B.

[62] Park S. C., Jeong H. J., Heo M., Shin J. H., Ahn J. H. (2021). Carbon
Nanotube-Based Ion-Sensitive Field-Effect Transistors with an On-Chip
Reference Electrode Toward Wearable Sodium Sensing. ACS Appl. Electron. Mater..

[63] Bakker E., Bühlmann P., Pretsch E. (1997). Carrier-Based Ion-Selective Electrodes
and Bulk Optodes. 1. General Characteristics. Chem. Rev..

[64] Umezawa Y., Bühlmann P., Umezawa K., Tohda K., Amemiya S. (1963). Potentiometric
Selectivity Coefficients of Ion-Selective Electrodes. Part I. Inorganic
Cations (Technical Report). Pure Appl. Chem..

[65] Cao A., Mescher M., Bosma D., Klootwijk J. H., Sudhölter E. J.
R., Smet L. C. P. M. D. (2015). Ionophore-Containing
Siloprene Membranes: Direct Comparison between Conventional Ion-Selective
Electrodes and Silicon Nanowire-Based Field-Effect Transistors. Anal. Chem..

