# Supplementary material for: Through-Layer Contact with Encapsulated Graphene Using One-Step Laser Scribing
Source: ACS Appl Mater Interfaces. 2025 Aug 5;17(33):47474–86. doi: 10.1021/acsami.5c09559 (PMC12372793; doi:10.1021/acsami.5c09559)
Supplement: Supplementary file 1 [file am5c09559_si_001.pdf]

Supporting Information

Through-layer Contact with Encapsulated Graphene Using One-Step Laser Scribing

Sang-Chan Park[1], *Won Gyun Park*[1] *Chulsoo Kim*[1] *and Jae-Hyuk Ahn*[1*]

[1]Department of Electronic Engineering, Chungnam National University, Daejone 34134, Republic of Korea

[*]Correspondence to J.-H. Ahn (E-mail: jaehyuk@cnu.ac.kr)

Table of contents:

1. CO_2 laser average fluence calculation

2. Optimization of through-layer LIG formation through the PVC encapsulation layer

3. CO_2 laser multiscribing for etching thick encapsulation layers

4. Raw I–V data for organic and inorganic encapsulated through-layer contact devices

5. Optical top-view images of organic and inorganic encapsulated through-layer contact devices

6. Effects of O_2 plasma treatment on the encapsulated graphene layer

7. Encapsulation crack adjacent to the through-layer LIG

8. Fourier transform infrared spectroscopy (FT-IR) of various organic encapsulation layers and through-layer LIG

9. Raman spectroscopy of through-layer LIG with various organic encapsulation layers

10. X-ray photoelectron spectroscopy (XPS) of various inorganic encapsulation layers and through-layer LIG

11. Graphene transfer for multilayered electrical contacts based on Parylene C encapsulation layer

12. Transmission electron microscopy (TEM) images and energy-dispersive spectroscopy (EDS) mapping of Al_2O_3/Al encapsulation stacks

13. Na sensing mechanism using a Na-selective membrane

14. Electrical Na sensors based on conventional metal contacts

15. Comparison of the performance of the graphene transistor with that of previously reported devices

16. Comparison of the performance of the sodium sensor with that of previously reported ion-selective electrodes and ion-sensitive field-effect transistors

17. Selectivity coefficients of the sodium-selective membrane

1. CO_2 laser Average Fluence Calculation

In this study, the average fluence (F, J/cm^2) was calculated based on the average laser power and scanning speed of the CO_2 laser system. The system was operated in the quasi-continuous wave mode, delivering laser energy as discrete pulses during continuous scanning. The average fluence per pulse is defined as the energy delivered per unit area:

$$F = \frac{E_{pulse}}{A} \tag{1}$$

where E is the single-pulse energy (J) and A is the exposure area (cm^2). For a Gaussian beam, the exposure area is approximated as $A=\pi r_0^2$, where r_0 is beam radius at the focal point of the lens.

Because E was not directly measured, it was derived from the average laser power (P_{avg}) and effective pulse repetition rate (f) using the following relationship:

$$E = \frac{P_{avg}}{f} \tag{2}$$

The effective pulse repetition rate depends on the scanning speed and number of pulses per unit length. In this setup, the number of pulses was controlled by the "*pulses per inch*" (PPI) parameter, and the scanning motion was defined by the scan speed v (in/s). Accordingly, the repetition rate can be expressed as

$$f = PPI \cdot v \tag{3}$$

Substituting into the average fluence equation yields

$$F = \frac{P_{avg}}{\mathrm{PPI} \cdot v \cdot \pi r_0^2} \qquad \textbf{Equation S1}$$

All fluence values are reported in J/cm^2. The beam radius was measured as 120 μm (converted to cm), and the scan speed and power were varied according to the experimental design. The PPI was fixed at 1000 for all experiments. Unit conversion was performed to ensure dimensional consistency.

2. Optimization of through-layer LIG formation through PVC encapsulation layer

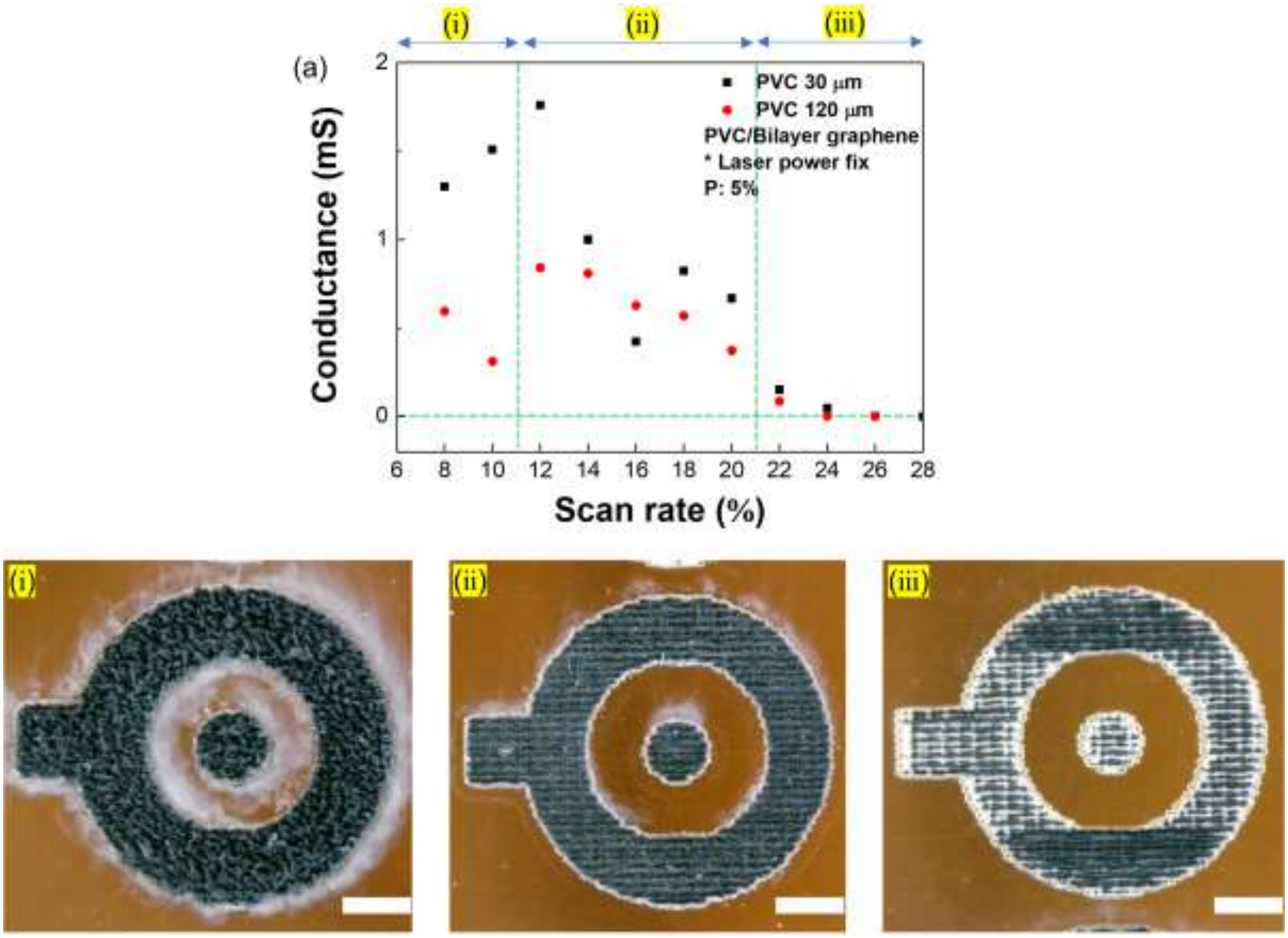

Figure S1. (a) Conductance of the through-layer LIG contact device under varying CO_2 laser scribing conditions, where the laser power was fixed while the laser scan rate was varied. Representative optical images for each region (i–iii) are presented. Scale bar: 1 mm. Scan rate: 8–10% (i); 12–20% (ii); 22–28% (iii).

3. CO_2 laser multiscribing for etching thick encapsulation layer

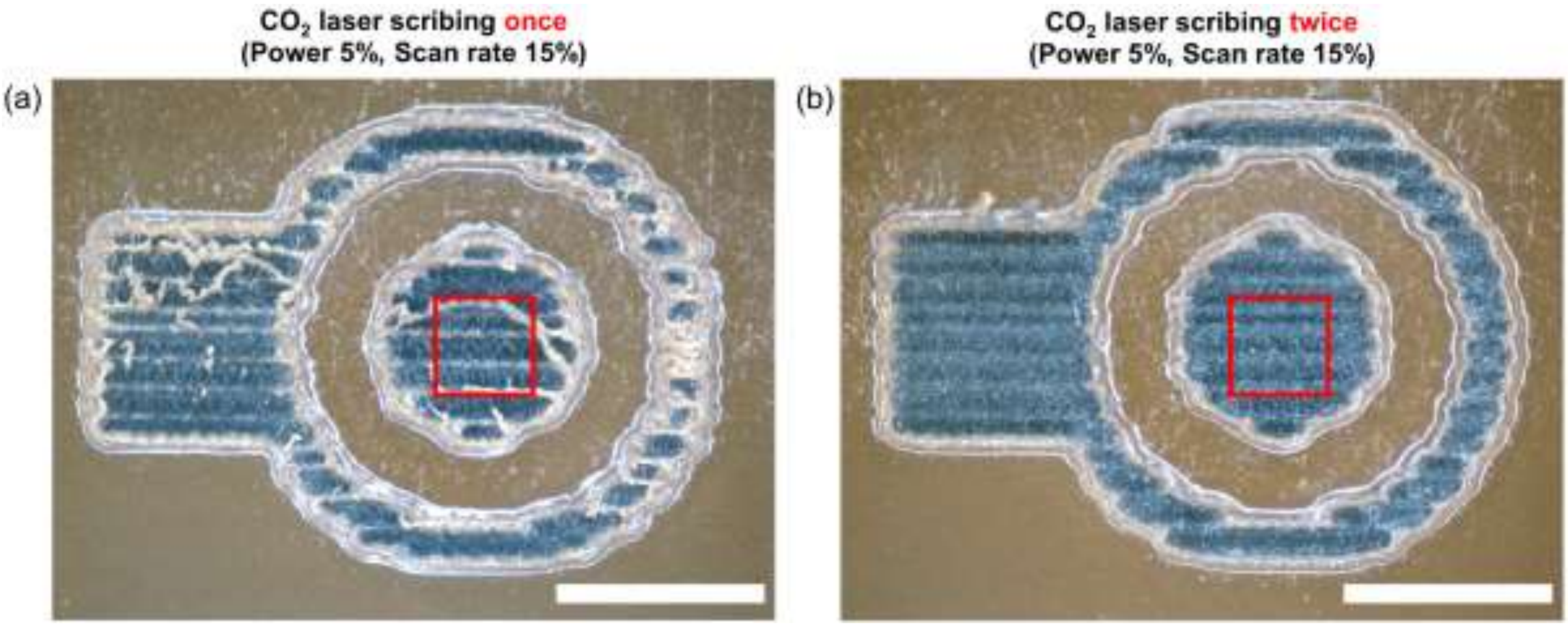

Figure S2. (a) Incomplete thick encapsulation layer etching and through-layer LIG formation via single CO_2 laser scribing. (b) Complete thick encapsulation layer etching and through-layer LIG formation with CO_2 laser scribing twice. Scale bar: 1 mm.

4. I-V raw data of the organic and inorganic encapsulated through-layer contact devices

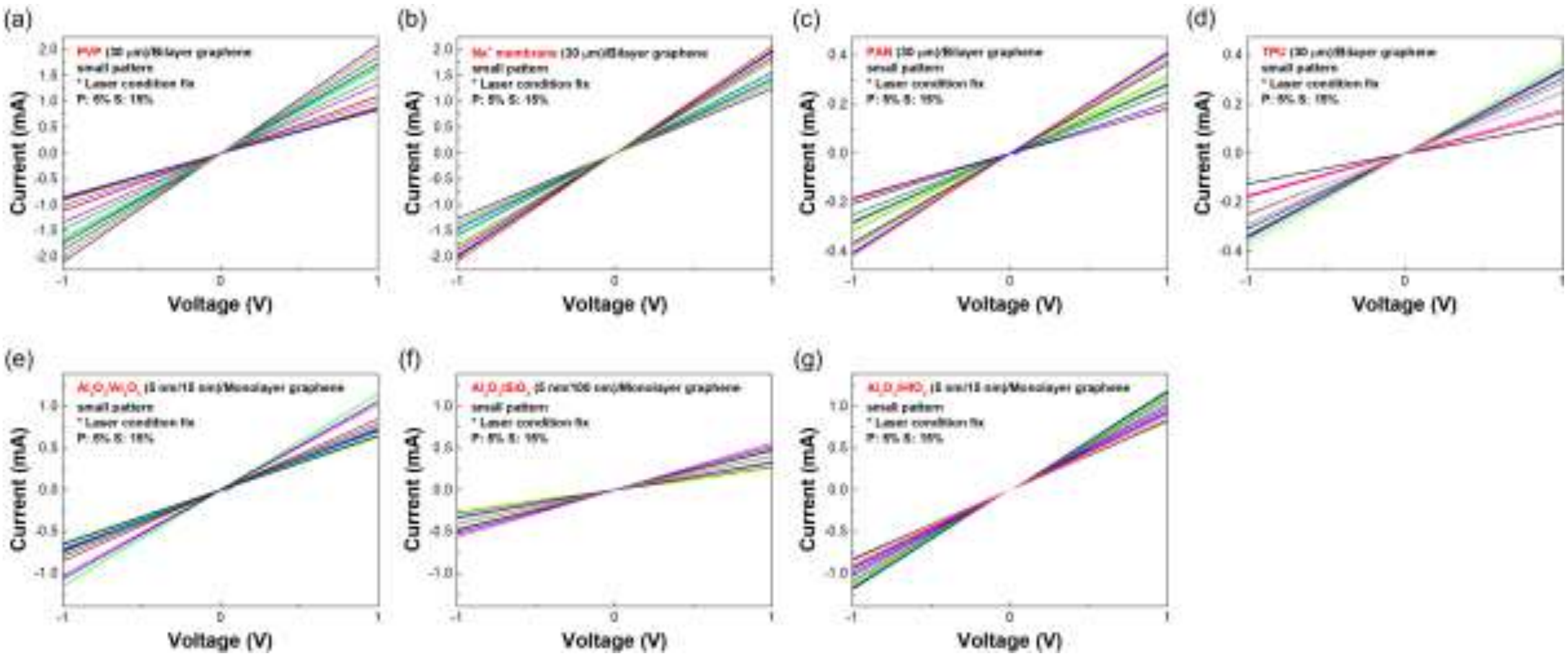

Figure S3. I–V raw data of through-layer contact devices with (a–d) organic encapsulation layers (PVP, Na^+ membrane, PAN, and TPU) and (e–g) inorganic encapsulation layers (Al_2O_3, SiO_2, and HfO_2).

5. Optical top view images of the organic and inorganic encapsulated through-layer contact devices

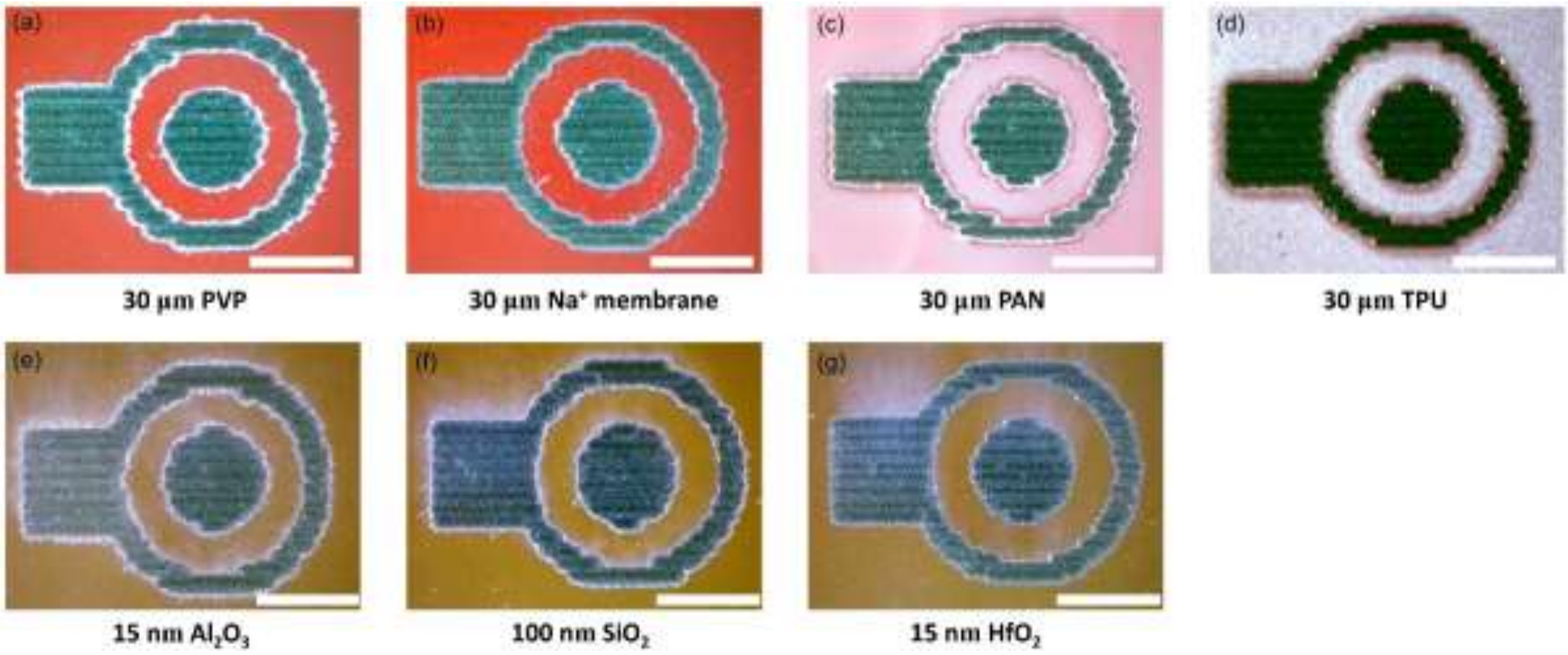

Figure S4. Optical top-view images of through-layer contact devices encapsulated with (a-d) organic layers (PVP, Na^+ membrane, PAN, and TPU) and (e-g) inorganic layers (Al_2O_3, SiO_2, and HfO_2).

6. Effects of O_2 plasma treatment to encapsulated graphene layer

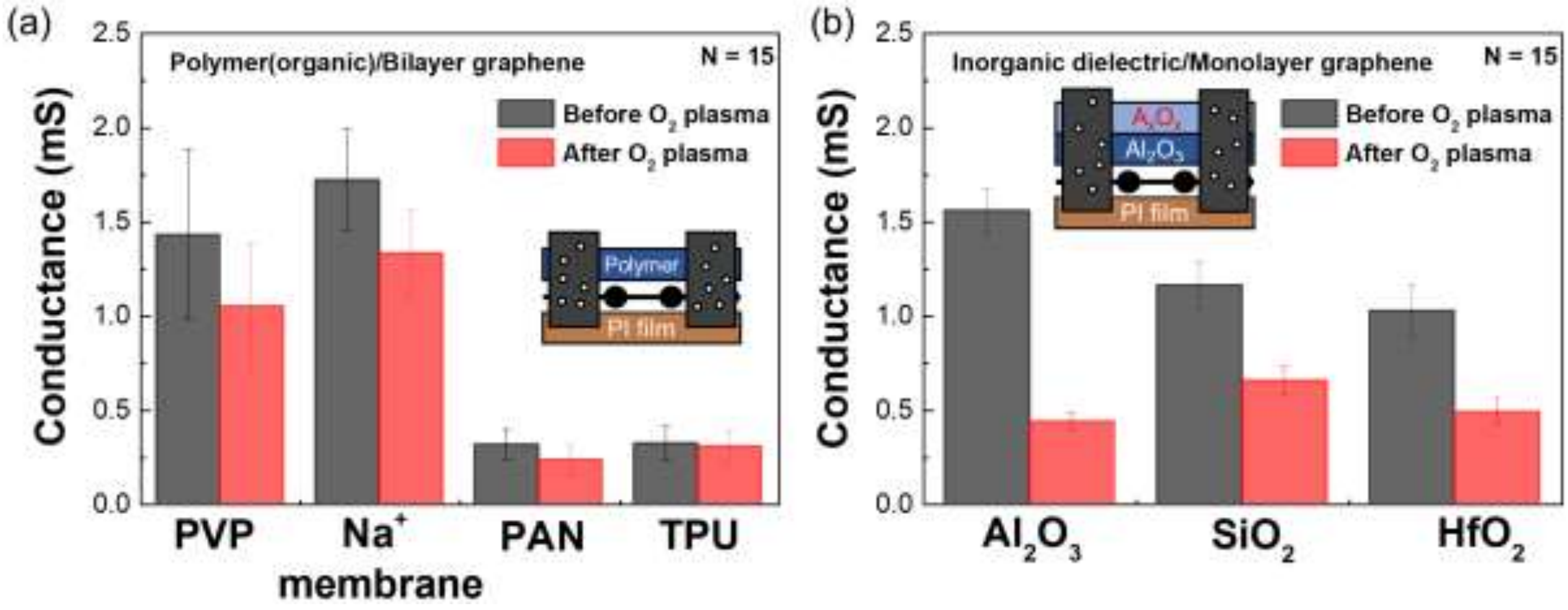

Figure S5. Conductance change of through-layer LIG contact devices with various organic (PVP, Na^+ membrane, PAN, and TPU) (a) and inorganic (Al_2O_3, SiO_2, and HfO_2) (b22) encapsulation layers after O_2 plasma treatment (100 W, 30 sccm, 50 mTorr, 5 min).

When the graphene layer was covered by organic and inorganic encapsulation layers, the electrical current between the through-layer LIG electrodes and graphene still flowed with a slight decrease after the O_2 plasma treatment [PVP (26.7%), Na^+ membrane (22.9%), PAN (26.1%), TPU (5.2%), Al_2O_3 (71.9%), SiO_2 (43.7%), and HfO_2 (52.4%)]. As expected, the encapsulation layer could withstand the O_2 plasma damage to the graphene layer. Because inorganic encapsulation layers generally have a higher melting point than organic encapsulation layers, the irradiation of CO_2 lasers to inorganic encapsulation layers creates more cracks at the edges of LIG electrodes, making graphene prone to O_2 plasma damage. These cracks and edges near the LIG for the organic and inorganic encapsulation layers were verified by SEM images (Figure S6).

7. Encapsulation crack adjacent to through-layer LIG

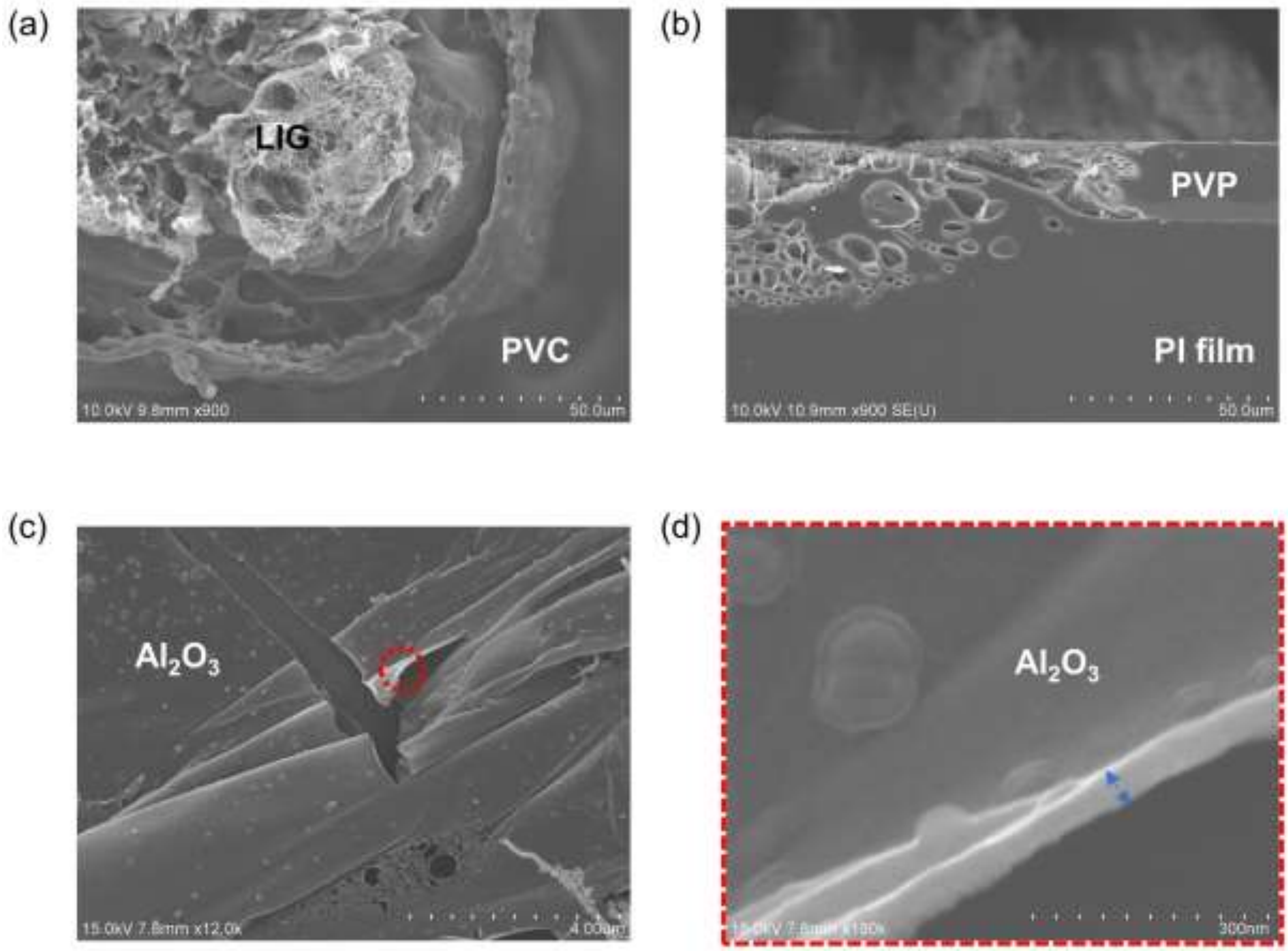

Figure S6. Edge region of through-layer LIG top view (a) and cross-sectional view (b) SEM images of the PVC and PVP encapsulation layers, respectively. (c) SEM image of through-layer LIG with an Al_2O_3 encapsulation layer. The red dotted circle indicates the region in the magnified SEM image (d).

8. Fourier-transform infrared spectroscopy (FT-IR) of various organic encapsulation layers and through-layer LIG

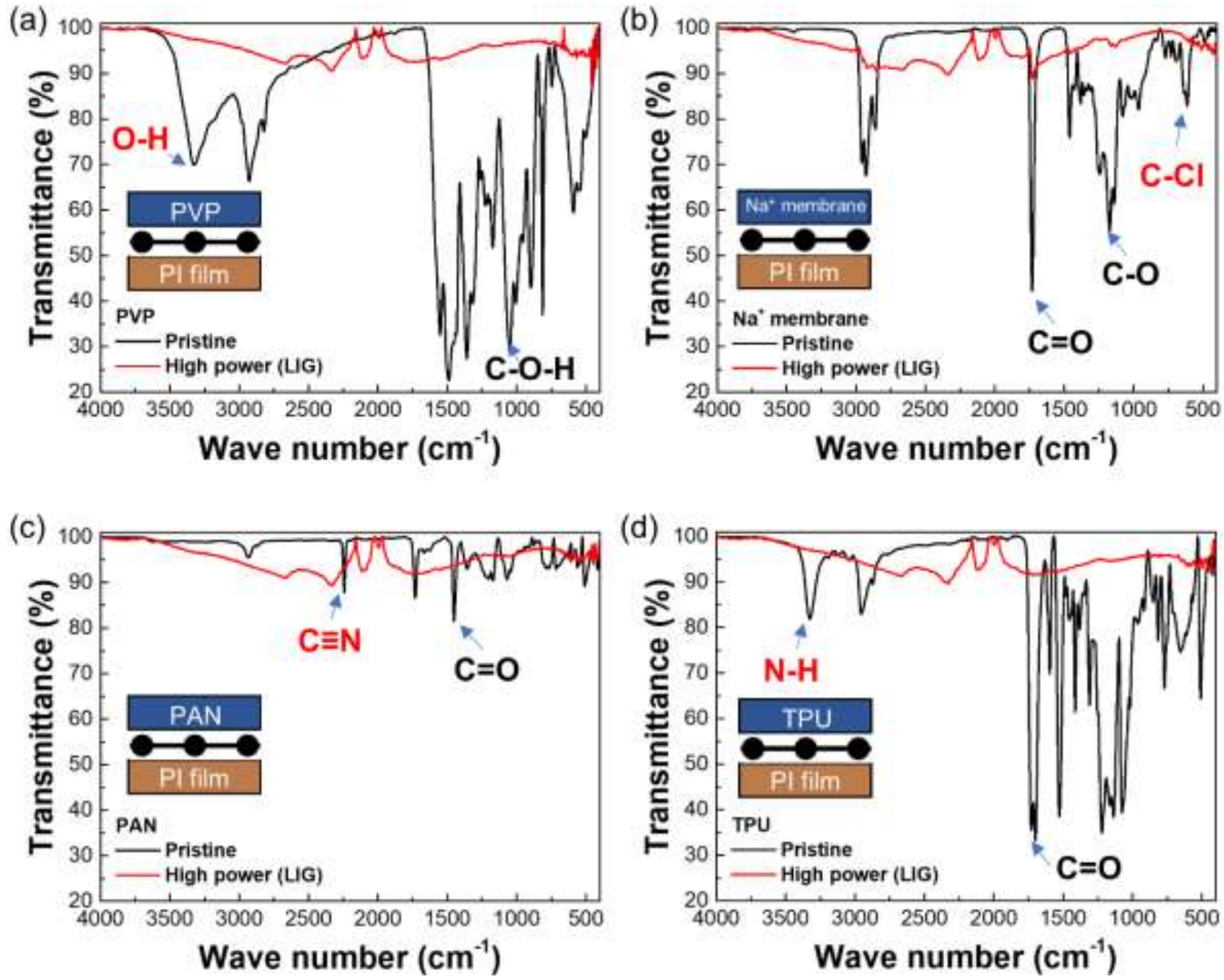

Figure S7. Transmittances of pristine organic encapsulation layers (PVP, Na+ membrane, PAN, and TPU) and LIG formed through them. In all graphs, the black and red lines represent each encapsulation layer and the corresponding LIG formed through them, respectively. The characteristic material peaks are highlighted in red: (a) hydroxyl groups of PVP, (b) vinyl chloride groups of PVC, (c) nitrile groups of PAN, and (d) amine groups of TPU.

Fourier-transform infrared spectroscopy (FT-IR) was performed to analyze the organic encapsulation layers and LIG formed through them. The pristine organic encapsulation layers

exhibited characteristic peaks, including the hydroxyl group at 3325 cm^{-1} for PVP [S1], the vinyl chloride group at 613 cm^{-1} for PVC (the matrix for the Na-selective membrane) [S2], the nitrile group at 2243 cm^{-1} for PAN [S3], and the amine group at 3325 cm^{-1} for TPU [S4]. The transmittance curves obtained from the FT-IR analysis varied depending on the chemical structure owing to molecular vibrations. However, all LIG structures formed using different organic encapsulation layers exhibited similar transmittance curves. When CO_2 laser scribing was conducted with sufficient energy, it effectively etched the organic encapsulation layers and facilitated LIG formation, resulting in the disappearance of the characteristic peaks corresponding to the pristine organic encapsulation layers and the emergence of broad bands associated with LIG. All the transmittance curves of LIG formed with various organic encapsulation layers exhibited a broad spectral range from 1650 cm^{-1} to 1740 cm^{-1}, corresponding to C=C and C=O stretching vibrations [S5].

9. Raman spectroscopy of through-layer LIG with various organic encapsulation layers

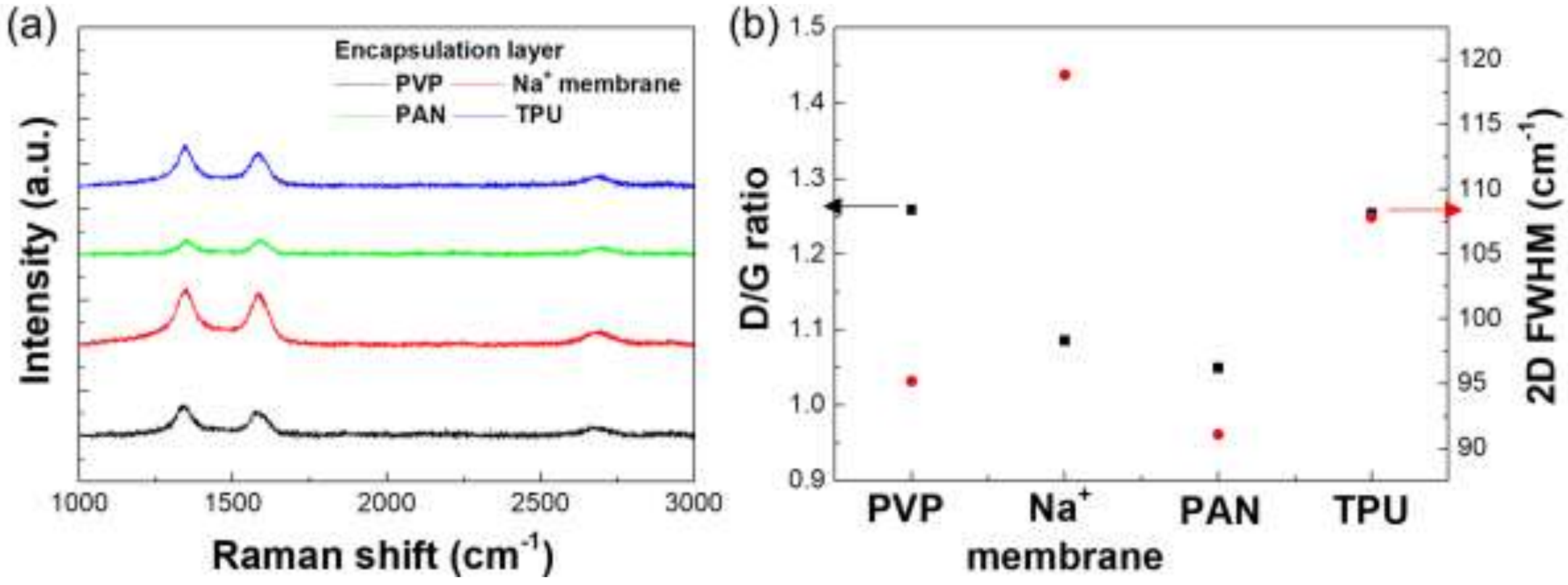

Figure S8. (a) Raman spectra of LIG formed using various organic encapsulation layers (PVP, Na^+ membrane, PAN, and TPU). (b) Extracted intensity ratio of the D/G peaks and full width at half maximum (FWHM) of the LIG 2D band.

The Raman spectrum of the through-layer LIG exhibited characteristic LIG peaks, including the D, G, and 2D bands. Irrespective of the type of organic encapsulation layer, all the through-layer LIG samples displayed similar Raman spectra. Specifically, the intensities of the D and G peaks are nearly identical, indicating a high density of defect sites in the through-layer LIG. The intensity ratios of the D and G peaks are greater than 1. According to our previous study, a lower scan rate resulted in a decreased D/G intensity ratio when no encapsulation layer was present [S6]. However, during CO_2 laser irradiation of the encapsulated graphene layer, some laser energy is consumed in etching the encapsulation layer, leading to incomplete LIG formation. This effect mimicked the impact of increasing the scan rate in the absence of an encapsulation layer, as both scenarios reduced the effective energy delivered to the graphene. Additionally, the intensity of the G band consistently exceeds that of the 2D band, indicating that LIG consists of multiple stacked layers [S7]. The full width at half-maximum (FWHM) of the 2D band exceeds 90 cm^{-1}, further supporting the

presence of multilayer graphene. According to Hao et al., the FWHM of the 2D band for single-layer graphene is 26.3 cm^{-1}, increasing to 52.1, 56.1, and 62.4 cm^{-1} as the number of graphene layers increases from bilayer to four layers [S8].

10. X-ray photoelectron spectroscopy (XPS) of various inorganic encapsulation layers and through-layer LIG

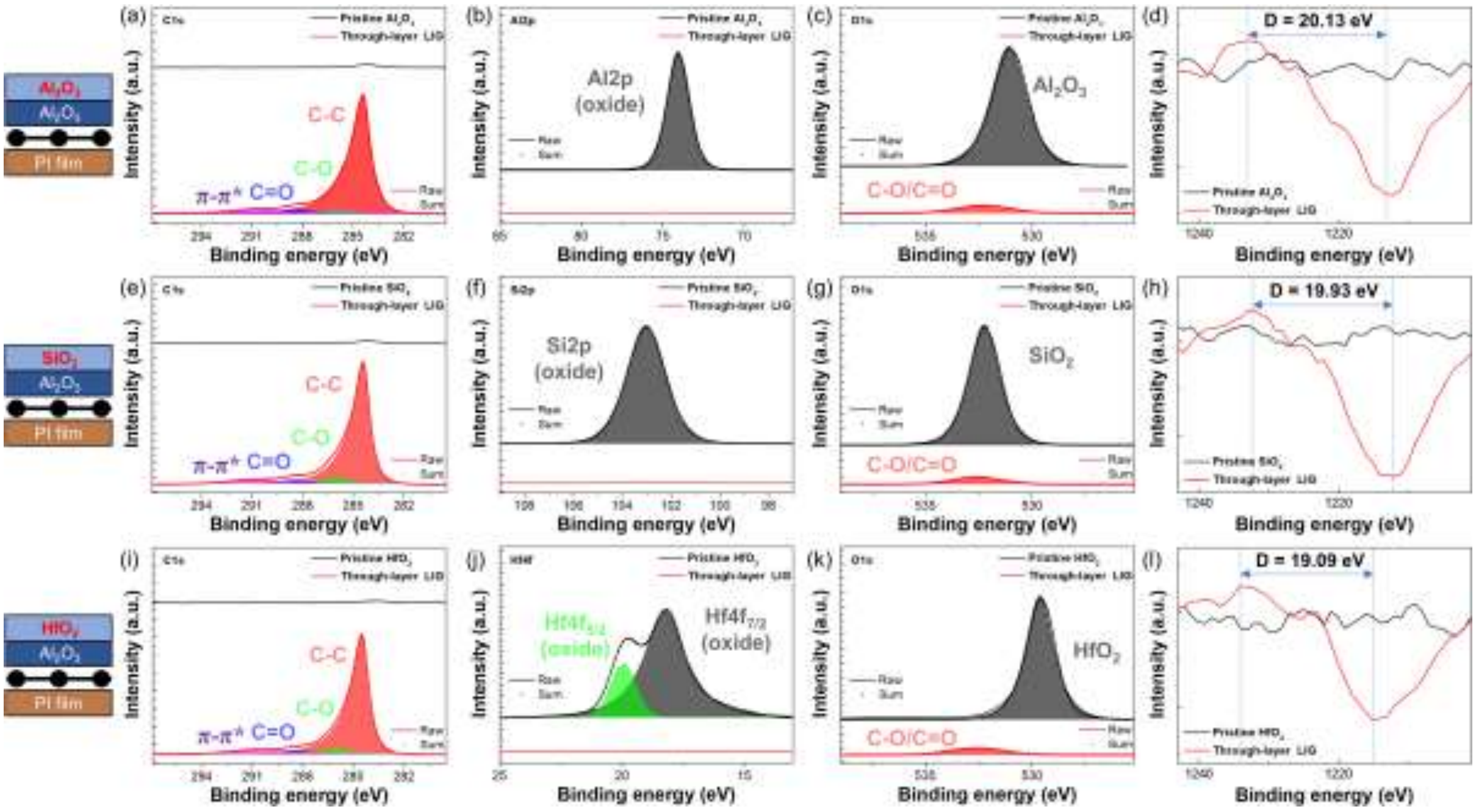

Figure S9. XPS analysis of the various inorganic encapsulation layers and LIG. C 1s spectrum of pristine Al_2O_3 (a), SiO_2 (e), and HfO_2 (i). Al 2p, Si 2p, and Hf 4f spectra of pristine Al_2O_3 (b), SiO_2 (f), and HfO_2 (j). O 1s spectra of pristine Al_2O_3 (c), SiO_2 (g), and HfO_2 (k). The differentiation of C *KLL* Auger regions and D parameter of pristine Al_2O_3 (d), SiO_2 (h), and HfO_2 (l). All spectra correspond to the through-layer LIG formed with various inorganic encapsulation layers, as indicated by the red line. On the left side of each row, the sample structure is described using a 2D schematic.

X-ray photoelectron spectroscopy (XPS) was conducted to analyze the pristine inorganic encapsulation layers (Al_2O_3, SiO_2, and HfO_2) and the LIG formed through them. In the C1s spectrum, the pristine inorganic encapsulation layers did not exhibit distinct peaks. However, following through-layer LIG formation via CO_2 laser scribing, four distinct peaks emerge at binding energies of approximately 284.4, 286, 288.5, and 291 eV, corresponding to C=C, C–

O, C=O, and π–π* transitions, respectively (Figure S9(a, e, i)). The presence of the π-π* transition indicates a high degree of graphitization in the LIG, which is primarily composed of sp^2-hybridized carbon [S9]. The pristine inorganic encapsulation layers were further characterized in the Al 2p, Si 2p, and Hf 4f spectral regions (Figure S9(b, f, j)). As expected, after through-layer LIG formation, all the characteristic peaks disappeared. Additionally, in the O1s spectrum (Figure S9(c, g, k)), peak shifts were observed owing to differences in the binding energy between the metalloid oxides, metal oxides, and carbon oxides, while the intensity reduction was attributed to the emergence of C–O, C=O peaks. Notably, LIG contained less oxygen than the pristine inorganic encapsulation layers. This confirms that the CO_2 laser-scribing process etched the inorganic encapsulation layers while simultaneously forming a through-layer LIG. Furthermore, the LIG primarily consists of sp^2-hybridized carbon, similar to highly ordered pyrolytic graphite (HOPG), as confirmed by the calculated D parameter values (20.13 eV for Al_2O_3, 19.03 eV for SiO_2, and 19.09 eV for HfO_2). These values were derived from the energy difference between the maximum and minimum intensities in the differentiated C *KLL* Auger spectra (Figure S9(d, h, l)) [S10].

11. Graphene transfer for multilayered electrical contacts based on Parylene C encapsulation layer.

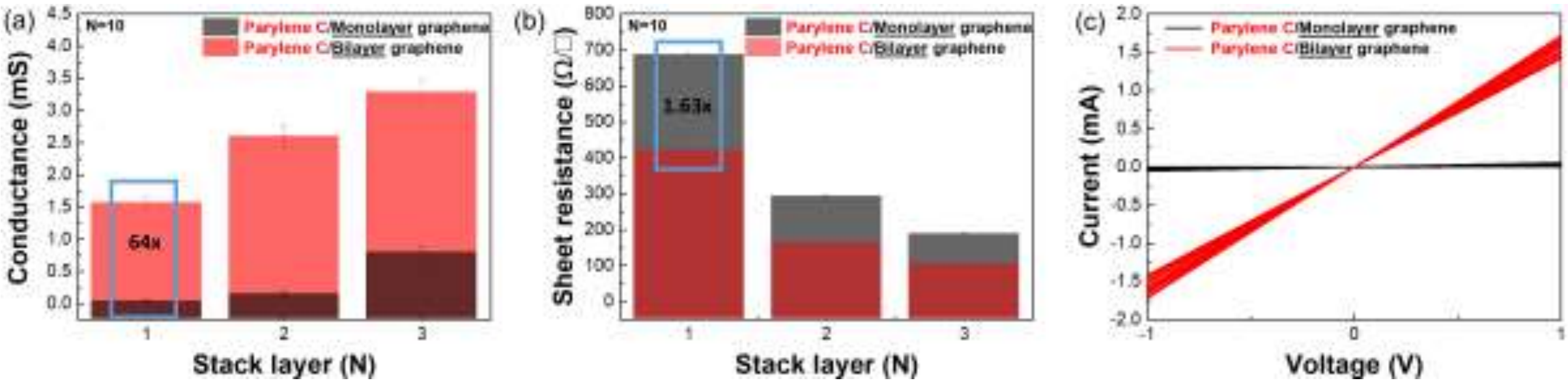

Figure S10. (a) Conductance of multilayer Parylene C/graphene stack extracted from circular-type through-layer LIG contact devices with monolayer and bilayer graphene. (b) Sheet resistance of the multilayer Parylene C/graphene stack measured by a noncontact eddy current probe using monolayer and bilayer graphene. (c) Raw I–V data for the Parylene C/graphene stack with monolayer and bilayer graphene.

12. Transmission electron microscopy (TEM) images and energy-dispersive spectroscopy (EDS) mapping of Al_2O_3/Al encapsulation stacks

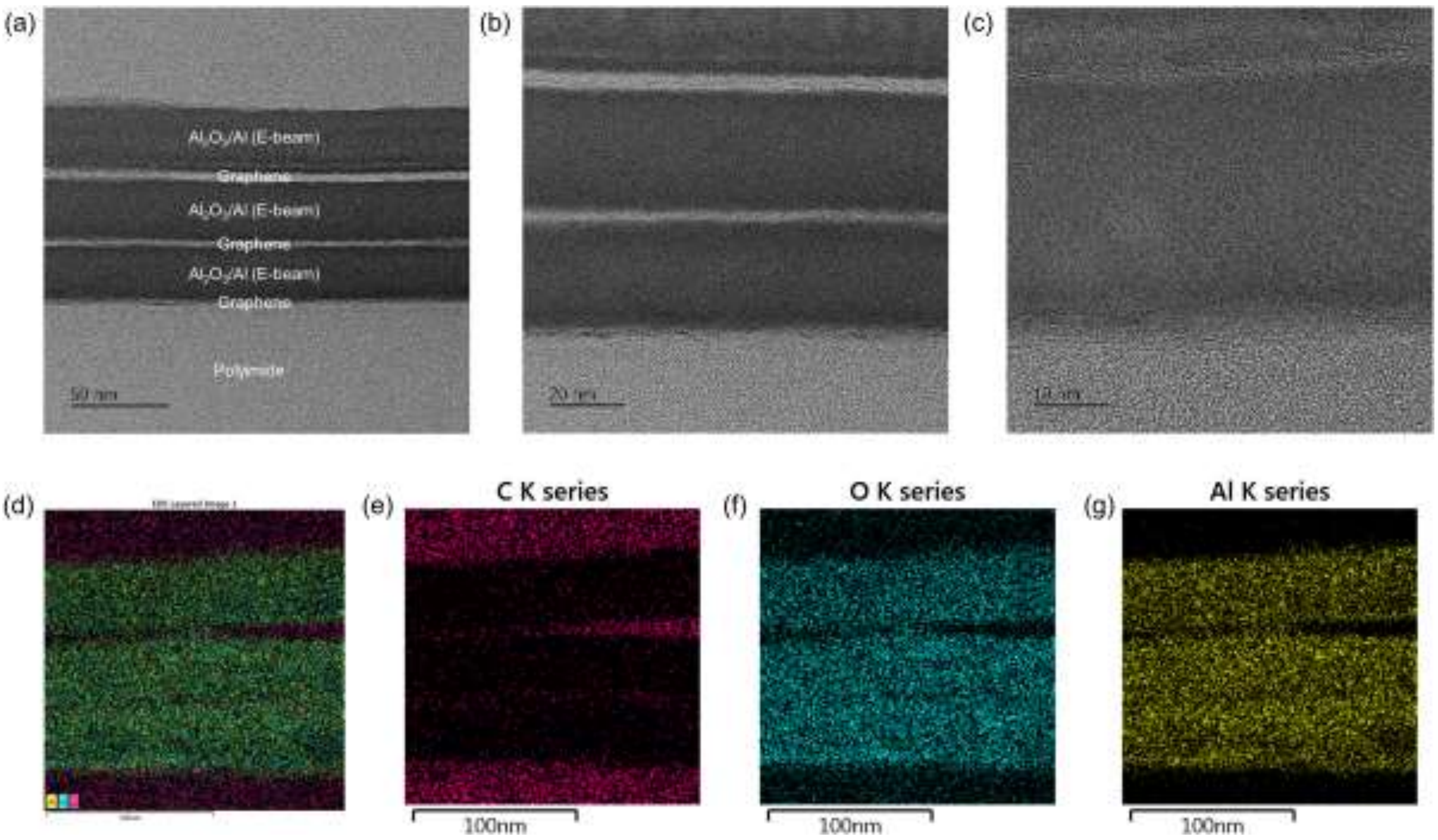

Figure S11. (a–c) TEM images of a three-stack structure consisting of Al_2O_3/Al/graphene on a PI film. (b, c) Magnified TEM images of the sample (a). (d, e) EDS mapping images of the three-stack Al_2O_3/Al/graphene structure on the PI film. (d) EDS mapping images of C, O, and Al. (e–g) Individual EDS mapping images of C, O, and Al.

13. Na sensing mechanism using a Na-selective membrane

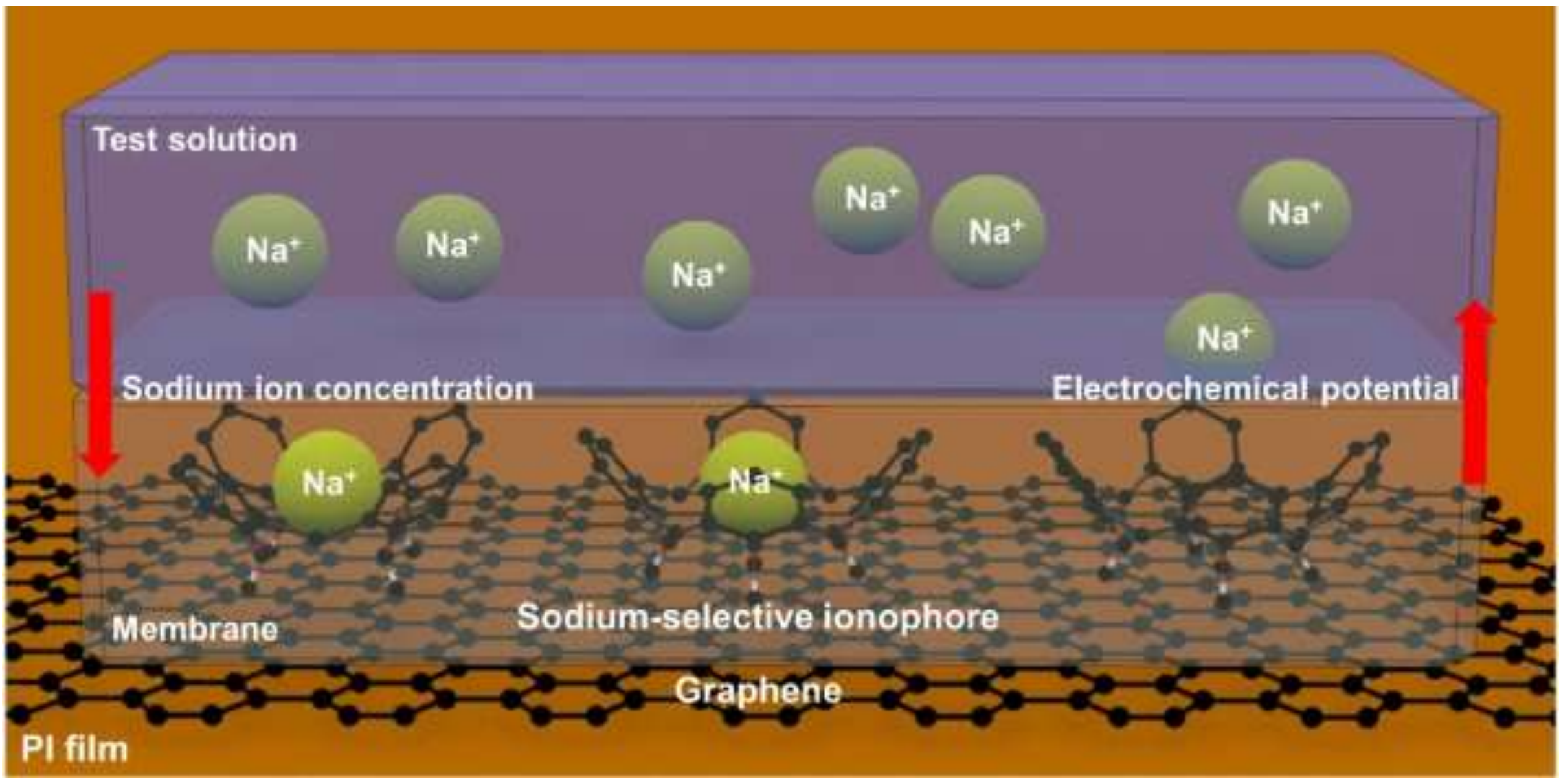

Figure S12. Graphical 3D schematic of the Na sensing mechanism, where the Na-selective ionophore acts as a capturing component for Na ions.

The Nernst equation is used to predict the equilibrium electrochemical potential (V_{Na}), which opposes the concentration difference gradient:

$$V_{Na} = \frac{RT}{zF} \ln \frac{[Na^+]_{out}}{[Na^+]_{in}}$$

where R is the gas law constant (8.31 J·K^{-1}·mol^{-1}), F is Faraday's constant (96,500 C·mol^{-1}), T is absolute temperature, z is the valance of the ion (+1 for Na ions), and $[Na^+]_{out}/[Na^+]_{in}$ represents the ratio of Na-ion concentrations in the test solution and the membrane, respectively [S11]. The theoretical Nernst limit at room temperature (300 K) calculated using this equation is 60 mV/decade. As the Na concentration increases, a positive electrochemical potential is generated, leading to a higher open-circuit potential.

When a graphene transistor with a Na-selective membrane is employed as a Na sensor, the

electrochemical potential contributes to the overall gate voltage applied via the reference electrode, shifting the transfer curve of the graphene transistor to the left. In addition, the sensitivity of the sensor in a mixed ionic background was lower than that in a solution containing only Na ions. According to the Debye–Hückel theory, higher ionic strength due to coexisting interfering ions at higher molarities reduces ion activity, thereby decreasing sensitivity [S12].

14. Electrical Na-sensor based on conventional metal contacts.

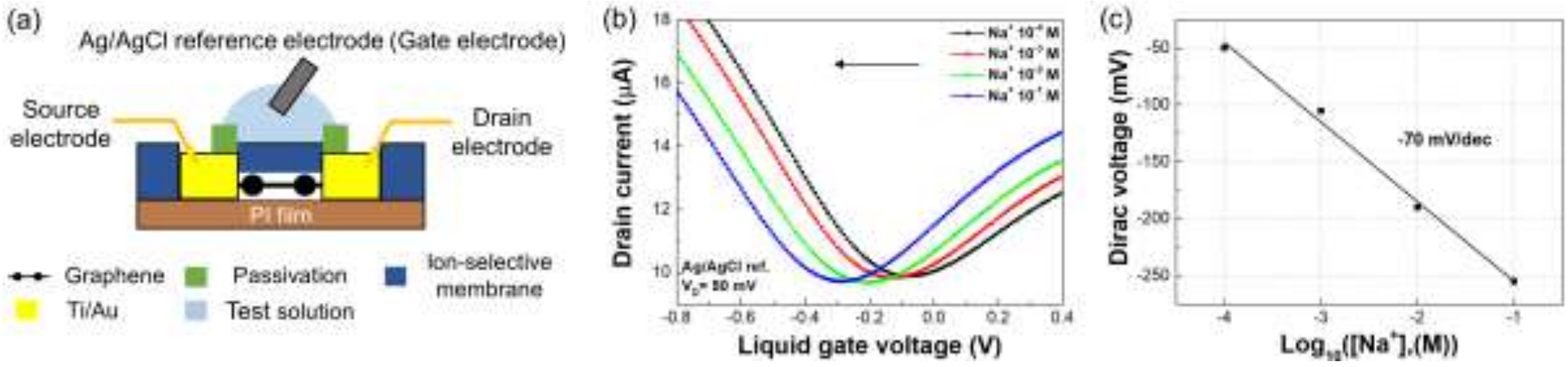

Figure S13. (a) 2D schematic of an electrical Na sensor with conventional Ti/Au (10/60 nm) source/drain electrodes in contact with the graphene layer. (b) Transfer curve of the electrolyte-gated graphene field-effect transistor, measured for Na-ion concentrations ranging from 0.1 mM to 100 mM. (c) Sensitivity of the electrical Na sensor across Na-ion concentrations from 0.1 mM to 100 mM. The calculated sensitivity is −70 mV/dec.

15. Comparison of the performance of the graphene transistor with that of previously reported devices

Table S1. Comparison of the performance of the graphene transistor with that of previously reported devices

Substrate	Gate dielectric	μ_h[cm^2/V·s]	μ_e[cm^2/V·s]	Reference
PET	Ion gel	30	50	[S13]
PET	Ion gel	852	452	[S14]
PET	Gel electrolyte	N/A	205	[S15]
Si	SiO_2	1104.6	N/A	[S16]
Si	SiO_2	563	1821 / 563	[S17]
Si	SiO_2	N/A	1750	[S18]
Parylene C	SiO_2	533.5	N/A	[S19]
PI	SiO_2/Al_2O_3	295	106	[S20]
PI	Al_2O_3/Al_2O_3	122	107	This work

N/A: not available or not applicable.

16. Comparison of the performance of the sodium sensor with that of previously reported ion-selective electrodes and ion-sensitive field-effect transistors

Table S2. Comparison of the performance of the sodium sensor with those of previously reported ion-selective electrodes and ion-sensitive field-effect transistors

Substrate	Channel or working electrode material	Sensitivity [mV dec^{-1}]		Reference
		Potentiometric (OCP)	Potentiometric (ISEFT)	
Fused silica	Graphene	N/A	49.2	[S21]
Si	s-SWCNT[a]	N/A	71.7	[S22]
Si	Si	N/A	60.7	[S23]
Glass	rrP3HT[b]	N/A	47 ± 7	[S24]
Paper	Gold	62.12	N/A	[S25]
PI tape or tattoo paper	MWCNT[c]	58	N/A	[S26]
Commercial tape	Carbon	35	N/A	[S27]
PI	Carbon	59.5	N/A	[S28]
PI	Graphene	52.5	68	This work

[a] Semiconducting single-wall carbon nanotube, [b] Regioregular poly(3-hexylthiophene), [c] Multiwall carbon nanotube, [d] N/A: not available or not applicable

17. Selectivity coefficients of the sodium-selective membrane

Table S3. Potentiometric selectivity coefficients (log K^{pot}) of the sodium-selective membrane for Na^+ over K^+, Ca^{2+}, and Mg^{2+}, determined by the separate solution method under equimolar 10 mM conditions.

Interfering ion	Log K^{pot} (Potentiometric, OCP)	Log K^{pot} (Potentiometric, ISFET)
K^+	−2.22	−2.79
Ca^{2+}	−2.68	−2.99
Mg^{2+}	−3.01	−4.02

References

[S1] Zhang, G.; Kim, C.; Choi, W. Poly(4-Vinylphenol) as a New Stable and Metal-Free Sensitizer of Titania for Visible Light Photocatalysis through Ligand-to-Metal Charge Transfer Process. *Catal. Today* **2017**, *281*, 109–116.

[S2] Park, E. J.; Park, B. C.; Kim, Y. J.; Canlier, A.; Hwang, T. S. Elimination and Substitution Compete During Amination of Poly(Vinyl Chloride) with Ehtylenediamine: XPS Analysis and Approach of Active Site Index. *Macromol. Res.* **2018**, *26* (10), 913–923.

[S3] Chennam, P. K.; Kachlík, M.; Říhová, M.; Čičmancová, V.; Maca, K.; Macak, J. M. Synthesis of Centrifugally Spun Polyacrylonitrile-Carbon Fibers. *J. Mater. Res. Technol.* **2024**, *28*, 2199–2205.

[S4] Tong, L.; Wang, X. X.; He, X. X.; Nie, G. Di; Zhang, J.; Zhang, B.; Guo, W. Z.; Long, Y. Z. Electrically Conductive TPU Nanofibrous Composite with High Stretchability for Flexible Strain Sensor. *Nanoscale Res. Lett.* **2018**, *13*.

[S5] Ţucureanu, V.; Matei, A.; Avram, A. M. FTIR Spectroscopy for Carbon Family Study. *Crit. Rev. Anal. Chem.* **2016**, *46* (6), 502–520.

[S6] Park, W. G.; Park, S. C.; Cho, H. J.; Oh, Y. W.; Kang, I. S.; Ahn, J. H. In Situ Electrical Contacts to Graphene by Laser Scribing. *ACS Appl. Nano Mater.* **2024**, *7* (9), 10952–10962.

[S7] Malard, L. M.; Pimenta, M. A.; Dresselhaus, G.; Dresselhaus, M. S. Raman Spectroscopy in Graphene. *Phys. Rep.* **2009**, *473* (5–6), 51–87.

[S8] Hao, Y.; Wang, Y.; Wang, L.; Ni, Z.; Wang, Z.; Wang, R.; Koo, C. K.; Shen, Z.; Thong, J. T. L. Probing Layer Number and Stacking Order of Few-Layer Graphene by Raman Spectroscopy. *Small* **2010**, *6* (2), 195–200.

[S9] Lamberti, A.; Perrucci, F.; Caprioli, M.; Serrapede, M.; Fontana, M.; Bianco, S.; Ferrero, S.; Tresso, E. New Insights on Laser-Induced Graphene Electrodes for Flexible Supercapacitors: Tunable Morphology and Physical Properties. *Nanotechnology* **2017**, *28* (17).

[S10] Mezzi, A.; Kaciulis, S. Surface Investigation of Carbon Films: From Diamond to Graphite. *Surf. Interface Anal.* **2010**, *42* (6–7), 1082–1084.

[S11] Wright, S. H. Generation of Resting Membrane Potential. *Am. J. Physiol. - Adv. Physiol. Educ.* **2004**, *28* (4), 139–142.

[S12] Debye, P.; Hückel, E. *On the Theory of Electrolytes. I. Freezing Point Depression and Related Phenomena*; **1923**.

[S13] Ning, J.; Wang, Y.; Feng, X.; Wang, B.; Dong, J.; Wang, D.; Yan, C.; Shen, X.; Wang, X.; Zhang, J.; Hao, Y. Flexible Field-Effect Transistors with a High on/off Current Ratio Based on Large-Area Single-Crystal Graphene. *Carbon* **2020**, *163*, 417–424.

[S14] Lee, S. K.; Humayun Kabir, S. M.; Sharma, B. K.; Kim, B. J.; Cho, J. H.; Ahn, J. H. Photo-Patternable Ion Gel-Gated Graphene Transistors and Inverters on Plastic. *Nanotechnology* **2014**, *25* (1).

[S15] Hong, N.; Kireev, D.; Zhao, Q.; Chen, D.; Akinwande, D.; Li, W. Roll-to-Roll Dry Transfer of Large-Scale Graphene. *Adv. Mater.* **2022**, *34* (3), 1–9.

[S16] Hung, Y. H.; Hsieh, T. C.; Lu, W. C.; Su, C. Y. Ultraclean and Facile Patterning of CVD Graphene by a UV-Light-Assisted Dry Transfer Method. *ACS Appl. Mater. Interfaces* **2023**, *15* (3), 4826–4834.

[S17] Kwon, B.; Bae, H.; Lee, H.; Kim, S.; Hwang, J.; Lim, H.; Lee, J. H.; Cho, K.; Ye, J.; Lee, S.; Lee, W. H. Ultrasensitive N-Channel Graphene Gas Sensors by Nondestructive Molecular Doping. *ACS Nano* **2022**, *16* (2), 2176–2187.

[S18] Belotcerkovtceva, D.; Panda, J.; Ramu, M.; Sarkar, T.; Noumbe, U.; Kamalakar, M. V.

High Current Limits in Chemical Vapor Deposited Graphene Spintronic Devices. *Nano Res.* **2023**, *16* (4), 4233–4239.

[S19] Park, D. W.; Kim, H.; Bong, J.; Mikael, S.; Kim, T. J.; Williams, J. C.; Ma, Z. Flexible Bottom-Gate Graphene Transistors on Parylene C Substrate and the Effect of Current Annealing. *Appl. Phys. Lett.* **2016**, *109* (15).

[S20] Nayfeh, O. M. Graphene Transistors on Mechanically Flexible Polyimide Incorporating Atomic-Layer-Deposited Gate Dielectric. *IEEE Electron Device Lett.* **2011**, *32* (10), 1349–1351.

[S21] Fakih, I.; Durnan, O.; Mahvash, F.; Napal, I.; Centeno, A.; Zurutuza, A.; Yargeau, V.; Szkopek, T. Selective Ion Sensing with High Resolution Large Area Graphene Field Effect Transistor Arrays. *Nat. Commun.* **2020**, *11* (1), 1–12.

[S22] Park, S. C.; Jeong, H. J.; Heo, M.; Shin, J. H.; Ahn, J. H. Carbon Nanotube-Based Ion-Sensitive Field-Effect Transistors with an On-Chip Reference Electrode Toward Wearable Sodium Sensing. *ACS Appl. Electron. Mater.* **2021**, *3* (6), 2580–2588.

[S23] Rovira, M.; Lafaye, C.; Wang, S.; Fernandez-Sanchez, C.; Saubade, M.; Liu, S. C.; Jimenez-Jorquera, C. Analytical Assessment of Sodium ISFET Based Sensors for Sweat Analysis. *Sens. Actuators B Chem.* **2023**, *393*, 134135.

[S24] Al Baroot, A. F.; Grell, M. Comparing Electron- and Hole Transporting Semiconductors in Ion Sensitive Water- Gated Transistors. *Mater. Sci. Semicond. Process.* **2019**, *89*, 216–222.

[S25] Cao, Q.; Liang, B.; Mao, X.; Wei, J.; Tu, T.; Fang, L.; Ye, X. A Smartwatch Integrated with a Paper-Based Microfluidic Patch for Sweat Electrolytes Monitoring. *Electroanalysis* **2021**, *33* (3), 643–651.

[S26] Roy, S.; David-Pur, M.; Hanein, Y. Carbon Nanotube-Based Ion Selective Sensors for Wearable Applications. *ACS Appl. Mater. Interfaces* **2017**, *9* (40), 35169–35177.

[S27] He, X.; Yang, S.; Pei, Q.; Song, Y.; Liu, C.; Xu, T.; Zhang, X. Integrated Smart Janus Textile Bands for Self-Pumping Sweat Sampling and Analysis. *ACS Sens.* **2020**, *5* (6), 1548–1554.

[S28] Vinoth, R.; Nakagawa, T.; Mathiyarasu, J.; Mohan, A. M. V. Fully Printed Wearable Microfluidic Devices for High-Throughput Sweat Sampling and Multiplexed Electrochemical Analysis. *ACS Sens.* **2021**, *6* (3), 1174–1186.
